# Interaction of Radiopharmaceuticals with Somatostatin Receptor 2 Revealed by Molecular Dynamics Simulations

**DOI:** 10.1021/acs.jcim.3c00712

**Published:** 2023-07-19

**Authors:** Silvia Gervasoni, Işılay Öztürk, Camilla Guccione, Andrea Bosin, Paolo Ruggerone, Giuliano Malloci

**Affiliations:** Department of Physics, University of Cagliari, Monserrato (Cagliari) I-09042, Italy

## Abstract

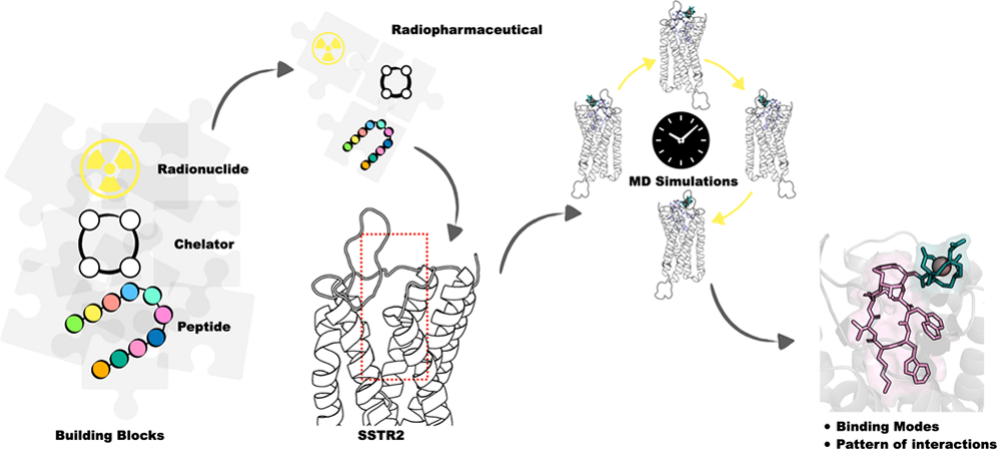

The development of drugs targeting somatostatin receptor 2 (SSTR2), generally overexpressed in neuroendocrine tumors, is focus of intense research. A few molecules in conjugation with radionuclides are in clinical use for both diagnostic and therapeutic purposes. These radiopharmaceuticals are composed of a somatostatin analogue biovector conjugated to a chelator moiety bearing the radionuclide. To date, despite valuable efforts, a detailed molecular-level description of the interaction of radiopharmaceuticals in complex with SSTR2 has not yet been accomplished. Therefore, in this work, we carefully analyzed the key dynamical features and detailed molecular interactions of SSTR2 in complex with six radiopharmaceutical compounds selected among the few already in use (^64^Cu/^68^Ga-DOTATATE, ^68^Ga-DOTATOC, ^64^Cu-SARTATE) and some in clinical development (^68^Ga-DOTANOC, ^64^Cu-TETATATE). Through molecular dynamics simulations and exploiting recently available structures of SSTR2, we explored the influence of the different portions of the compounds (peptide, radionuclide, and chelator) in the interaction with the receptor. We identified the most stable binding modes and found distinct interaction patterns characterizing the six compounds. We thus unveiled detailed molecular interactions crucial for the recognition of this class of radiopharmaceuticals. The microscopically well-founded analysis presented in this study provides guidelines for the design of new potent ligands targeting SSTR2.

## Introduction

Most efforts of modern medicine are addressed toward personalized medicine, in which each patient is treated according to the molecular features of the disease of interest.^1,2^ In this contest, radiopharmaceuticals have been extensively used to specifically target unhealthy tissues.^3,4^ According to the decay properties of the radionuclide, compounds can be employed for diagnostic or therapeutic purposes, or both (theranostics).^5^ Radionuclides emitting γ or β+ (*e.g.*, ^111^In and ^68^Ga) are exploited for imaging with single-photon emission computed tomography (SPECT) and positron emission tomography (PET), respectively, while those emitting β- or α (*e.g.*, ^177^Lu and ^211^At) are used for therapeutic treatments.^6^ In this last case, after the binding of a radiopharmaceutical to the given target and its subsequent internalization, a cytotoxic dose of radiation is delivered to the cancer cell.^7^ In some cases, the radionuclide emits both β+ and β- (^64^Cu), or γ and β- (^177^Lu), making their use suitable for theranostics.^4,8^

However, the inability to precisely quantify the radiation doses supplied to tumors and normal tissues has been one of the main drawbacks of radionuclide-based treatments. For example, ^111^In decays by electron capture emitting relatively high-energy γ photons with a half-life (*t*_1/2_) of 67.2 h, resulting in suboptimal imaging resolution and high radiation exposure in patients, which is even more pronounced when using short-lived isotopes, such as ^68^Ga (*t*_1/2_ = 1.13 h). Therefore, to alleviate this problem, it is possible to use a longer-lived radionuclide, allowing a more accurate assessment of biodistribution and tissue clearance. An example of alternative diagnostic agent is represented by the positron-emitting isotope ^64^Cu (*t*_1/2_ = 12.7 h, β+ = 17.4%, *E*_max_β+ = 653 keV).^9,10^ Both ^68^Ga and ^64^Cu are widely used in peptide receptor radionuclide therapy, a prominent example of which is represented by the treatment of neuroendocrine tumors (NET),^11^ where cancer cells are detected by exploiting a high concentration of somatostatin receptors on their surface.^12^ These receptors are class A G-protein-coupled receptors (GPCRs) and include the five distinct isoforms SSTR1–5.^13^ The isoform 2 (SSTR2), belonging to the SRIF1 sub-class together with the isoforms 3 and 5,^14^ is the most expressed in these types of tumors^15,16^ and, as a result, several drugs have been developed to specifically bind this receptor.^17^ To date, eight peptide-based radiopharmaceutical compounds targeting somatostatin receptors have been approved by FDA, and are routinely used in clinics for different applications (Table 1).^6^

**Table 1:** FDA Approved Radiopharmaceuticals for NET Diagnosis and Therapy, Their Targeted SSTR Isoform, and the Corresponding Applications^a^

Radiopharmaceutical	FDA date of approval	Targeted SSTR isoform^10^	Application
^111^In-pentetreotide	June 1994	2, 3, 5	SPECT imaging
^90^Y-DOTATOC	February 2002	2, 5	Therapy
^68^Ga-DOTATATE	June 2016	2	PET imaging
^177^Lu-DOTATATE	May 2018	2	Therapy
^68^Ga-DOTATOC	August 2019	2, 5	PET imaging
^64^Cu-SARTATE	May 2020	2	PET imaging
^64^Cu-DOTATATE	September 2020	2	PET imaging

a SPECT: single-photon emission computed tomography; PET: positron emission tomography. Data from Ref (18).

**Figure 1:**
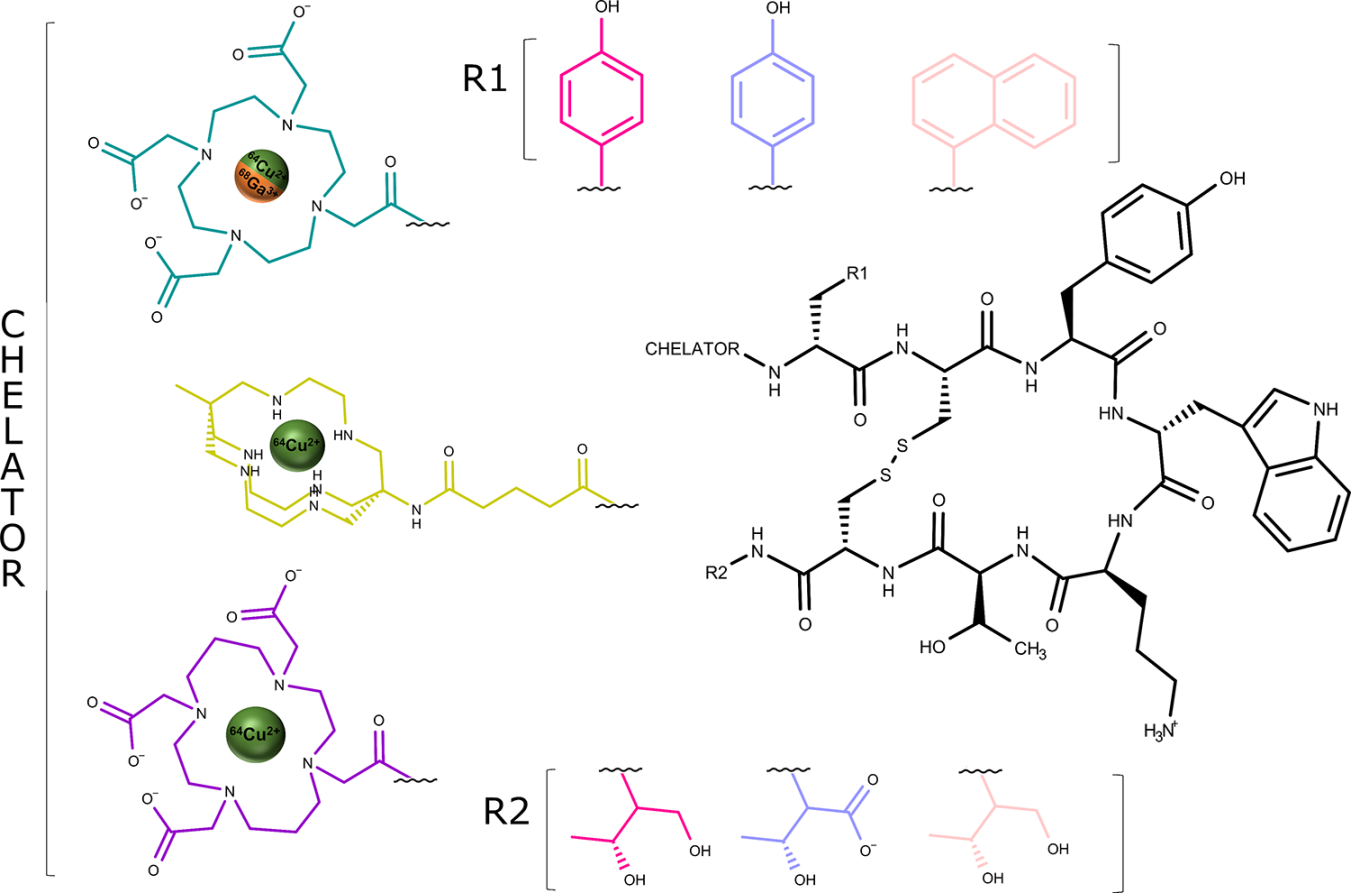
Schematic representation of the radiopharmaceuticals targeting SSTR2 investigated in this work. The two radionuclides (^68^Ga^3+^ in dark orange, ^64^Cu^2+^ in dark green), the structures of the three chelators (DOTA in dark cyan, SAR in lime, TETA in violet), and the three octreotide-based peptides (TOC in magenta, TATE in lilac, and NOC in pink) are reported.

Radiopharmaceuticals targeting SSTR2 share a similar three components structure made by (1) a biovector mimicking the structure of the endogenous ligand somatostatin, that is conjugated with (2) a chelator moiety carrying (3) a radionuclide (Figure 1).^19^

In the last year, different structures of SSTR2 in multiple conformational states have been published.^15,20,21,13,22,23^ Noteworthy, most of these structures are in complex with agonist ligands, which is often somatostatin or its analogous, like the octa-peptide octreotide. Both experimental and computational studies explored the conformational features of SSTR2 that are common to those of other class A GPCRs,^24^ and the key elements characterizing the binding with different types of ligands (see Figure S1 for an overview of the three-dimensional structure of SSTR2 and its main domains).^25^ The availability of such structural data can burst the development of new SSTR2 ligands able to bind the receptor with high affinity.^26^ However, a detailed molecular and atomistic-level description of the interaction of the radiopharmaceutical/SSTR2 complex is missing, thus hampering the rational design of new effective ligands of this family. Therefore, exploiting the available structural knowledge, in this work, we carefully analyzed the key dynamical features and detailed interactions of SSTR2 in complex with six radiopharmaceuticals. We focused on compounds loaded with either ^68^Ga or ^64^Cu: the former is the leading β+ emitting radiometal for PET imaging and is contained in two approved drugs (^68^Ga-DOTATATE and ^68^Ga-DOTATOC, Table 1), the latter is used for theranostic purposes and is contained in two approved drugs as well (^64^Cu-SARTATE and ^64^Cu-DOTATATE, Table 1). The two radionuclides were simulated in complex with three different chelators: 1,4,7,10-tetraazacyclododecane-*N*,*N*′,*N*″,*N*‴-tetraacetic acid (DOTA), 1,4,8,11-tetraazacyclotetradecane-1,4,8,11-tetraacetic acid (TETA), and 3,6,10,13,16,19-hexazabicyclo[6.6.6]icosane (SAR) (Figure 1). For the peptide portion, we considered three derivatives of octreotide, namely, TOC, TATE, and NOC: the first one is octreotide with the replacement of F3 into Y3, the second differs from TOC at the last residue (threonine T8 instead of threoninol T-ol8), and the third is octreotide with the replacement of F3 with naphthalene (Nal3) (Figure 1).

The choice of the radiopharmaceuticals was driven by the aim of exploring the influence of the different portions of the ligands in the interaction with the receptor by (1) considering the same radionuclide-chelator (^68^Ga-DOTA) and changing the peptide (TOC, TATE, and NOC), (2) considering the same chelator-peptide (DOTA-TATE) and changing the radionuclide (^68^Ga and ^64^Cu), and (3) considering the same radionuclide-peptide (^64^Cu-TATE) and changing the chelator (DOTA, TETA, SAR). Through multicopy μs-long molecular dynamics (MD) simulations based on a previous investigation on SSTR2 in different states,^25^ here we found analogies and differences in the interaction patterns characterizing the binding of the six compounds with SSTR2, and we discovered how each moiety can influence the dynamical behavior of the complexes. The detailed molecular-level analysis presented in this study, thoroughly mapping the SSTR2/ligand interactions, revealed previously unknown structural and mechanistic insights into molecular recognition processes of radiopharmaceuticals at SSTR2.

## Results and Discussion

We performed multicopy all-atom MD simulations of six metal-based radiopharmaceutical compounds in complex with SSTR2 (total simulation time of 15 μs per system). We focused on the influence that each component exerts on the dynamic properties of the complexes and the resulting interaction pattern. In the following, we analyze the role of the peptide moiety, the radionuclide, and the chelator by changing only one component at a time and comparing the MD results in terms of dynamics and detailed molecular interactions. Following this strategy, the role of the different components in the interaction could be evaluated more accurately. The Ballesteros-Weinstein numbering scheme for class A GPCRs is adopted throughout the paper.^27^ For better clarity, SSTR2 and ligand residues are indicated using the three- and one-letter nomenclature, respectively.

Generally, according to root-mean-square deviation (RMSD) of the ligand heavy atoms with respect to the initial frame of the production run, all compounds were highly stable inside the binding pocket, following the order ^64^Cu-DOTATATE (2.4 ± 0.1 Å) > ^68^Ga-DOTATOC (2.7 ± 0.2 Å) > ^64^Cu-TETATATE (3.3 ± 0.2 Å) = ^68^Ga-DOTATATE (3.3 ± 0.2 Å) > ^68^Ga-DOTANOC (3.6 ± 0.2 Å) > ^64^Cu-SARTATE (3.8 ± 0.4 Å) (Figure S2). As expected, most fluctuations were found at the terminal portions of the ligands (*i.e.*, the chelator moiety and the last residue of peptide T8 or T-ol8, Figure S3). Overall, for all compounds, we found the known conserved interactions involving residues located in the bottom part of the binding pocket of SSTR2 (*i.e.*, Asp122^3.32^, Gln126^3.36^) and the ^D^W4 and K5 motif of the ligands (Figure S4), as well as other residues already reported in previous works.^13,20,25^

In the following, we focus only on the comparative analysis of protein–ligand interactions characterizing the selected radiopharmaceuticals under investigation.

### Small Changes in the Peptide Structure Strongly Affect the Dynamics of the Complex

**Figure 2:**
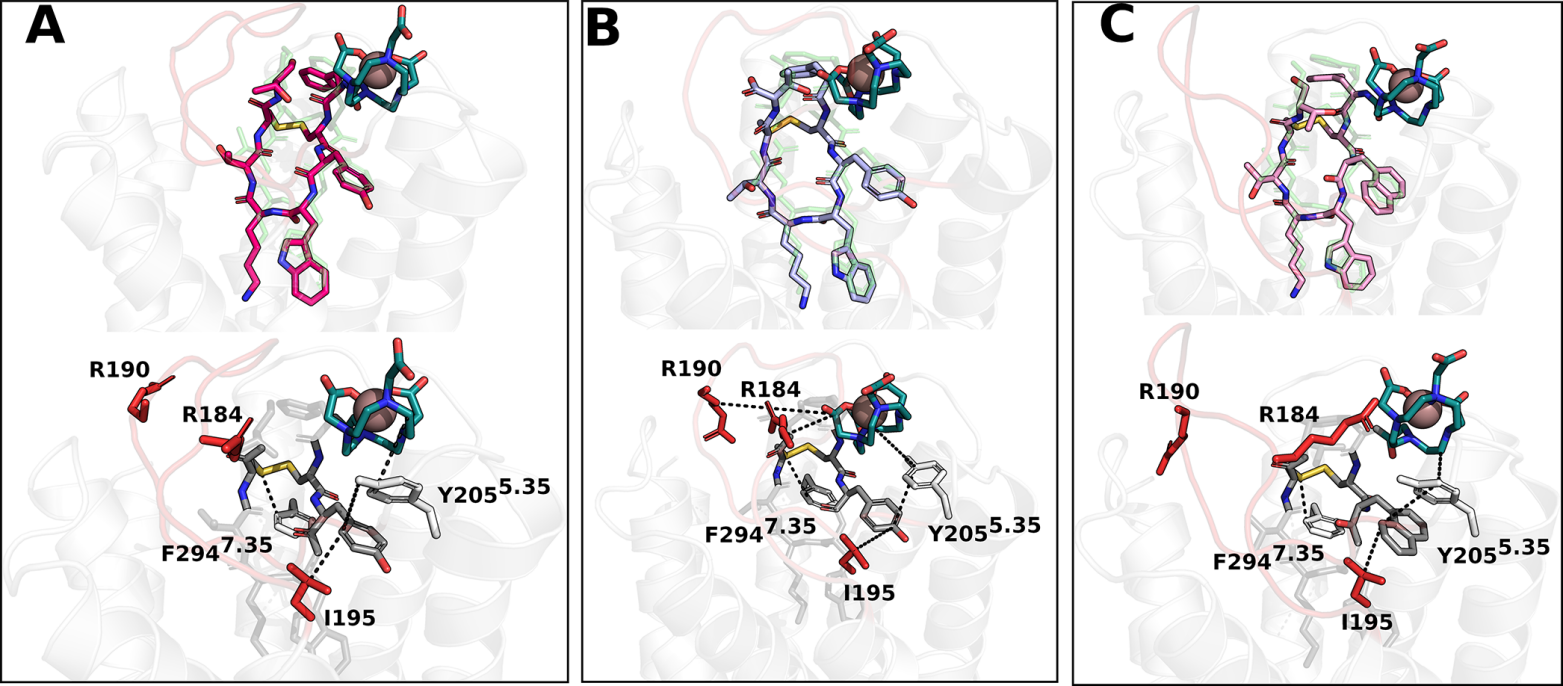
Representatives of the most populated conformational cluster extracted from MD trajectories for (A) ^68^Ga-DOTATOC, (B) ^68^Ga-DOTATATE, and (C) ^68^Ga-DOTANOC. The corresponding cluster populations are 57.3%, 67.3%, and 78.0%, respectively (Table S1). The extracellular loop ECL2 is colored in red; the gallium ion is represented as a pink sphere; the receptor is represented as a transparent white cartoon; the chelator DOTA is colored in dark cyan; the peptide portions TOC, TATE, and NOC are colored in magenta, violet, and pink, respectively. The top panels report the superimposition of the representative structural clusters with octreotide (green transparent sticks), taken from the PDB 7T11. In the bottom panels, the main interactions are shown as black dotted lines.

In the MD simulations of ^68^Ga-DOTATOC/TATE/NOC, we did not change the chelator-radionuclide portion (^68^Ga-DOTA), but only the peptide biovector (TOC, TATE, NOC). As a result, we were able to focus on the influence that small variations in the peptide structure (T8, T-ol8, Y3, or Nal3) exert on the interaction with SSTR2. In all cases, cluster analysis of MD trajectories (see the Computational Details section and Table S1) reveals a dominating cluster (population in the range of 60–80%) that does not differ significantly from the other two (RMSD in the range of 0.9–4.7 Å), confirming the overall stability of the binding modes (Table S1). Inspecting how the population of the dominant binding mode changes with time in all replicas, we found that it is the most populated one along the whole μs-long time-scale simulation or starting from a few hundreds of ns (see Figure S5). The representatives of the most populated clusters for the three cases are shown in Figure 2. Interestingly, the peptide portion overlaps neatly with the cryo-EM conformation of octreotide in complex with SSTR2 (RMSD octreotide vs TOC/TATE/NOC portions: 1.3/1.4/1.5 Å).^20^

**Figure 3:**
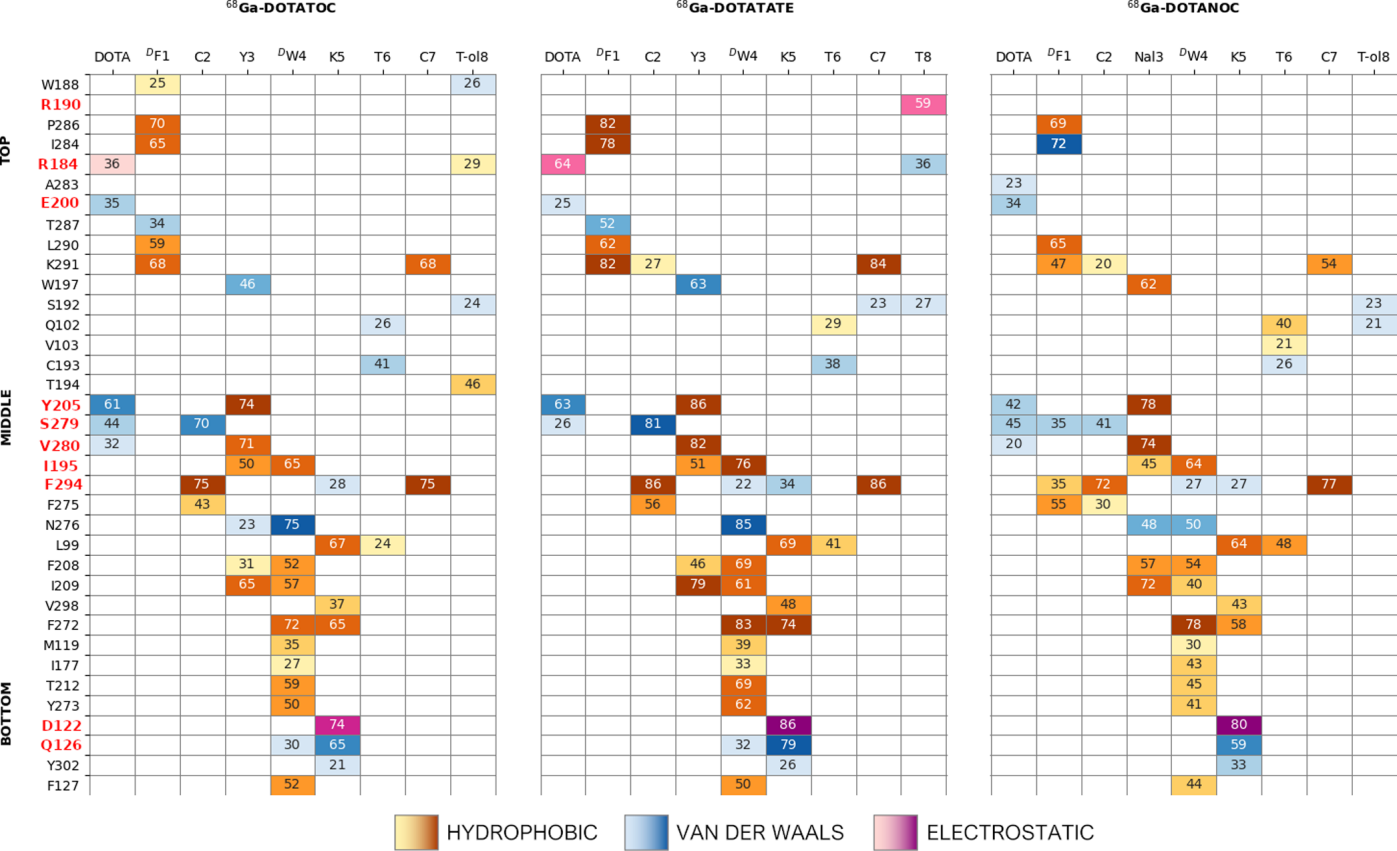
Interaction fingerprint analysis associated with SSTR2 residues distributed along the bottom, middle, and top portions of the binding pocket. The values inside the cells report the persistence of the interaction (%) between SSTR2 and ^68^Ga-DOTATOC (left), ^68^Ga-DOTATATE (middle), and ^68^Ga-DOTANOC (right). Residues discussed in the text are highlighted in red (R190, R184, E200, Y205, S279, V280, I295, F294, D122, and Q126). The cells are colored according to the different types of interactions: yellow to brown (low to high values of persistence) = hydrophobic, blue = van der Waals contact, and pink to purple (low to high values of persistence) = electrostatic. For the sake of clarity, only values above 20% are reported, and only the highest value associated with each ligand residue is shown. The full fingerprint heatmap is reported in Supporting Information (Figure S7A–C).

Looking at the structures, we found in all cases that the ^68^Ga-DOTA moiety was placed between TM5 and TM6, with some of the contacts involving ECL2 and ECL3 as well, while T8/T-ol8 interacted only with ECL2 (Figures 2 and S6). By combining the clustering of MD trajectories with the interaction fingerprint analysis (see the Computational Details section), we could identify detailed protein–ligand interactions stabilizing the complexes (Figure 3).

First, we discovered the prominent role of residue Tyr205^5.35^ in interacting with the ^68^Ga-DOTA moiety for 61, 63, and 42% of the total simulation time in ^68^Ga-DOTATOC/TATE/NOC, respectively. In addition, the binding of ligands is stabilized by hydrophobic interactions of Y3/Nal3 with Tyr205^5.35^ (persistence of 74, 86, and 78% for the three compounds, respectively). In turn, the ligand Y3/Nal3 residue interacts also with Ile195 (50, 51, and 45%) and Val280^6.59^ (71, 82, and 74%) (Figure 3). Interestingly, previous works reported the key role of Tyr205^5.35^ and Ile195 in the interaction of SSTR2 with SST14 and octreotide (through F7 and F3, respectively), and it was also pointed out that these two residues can contribute to the selectivity of the different SSTR isoforms.^20,21,13,23,28^ Furthermore, Phe294^7.35^ and Ser279^6.58^ (belonging to the hydrophobic sub-pocket constituted by TM6–7 and ECL3^13^) seem to be involved in isoform selectivity as well,^23^ and we found consistently their interaction with the disulfide bridge featured by all compounds (75/70% for ^68^Ga-DOTATOC, 86/81% for ^68^Ga-DOTATATE, 77/41% for ^68^Ga-DOTANOC). Although the aforementioned interactions are conserved, we noticed some differences in the persistence of the DOTA-Tyr205^5.35^ interaction. In particular, the replacement of tyrosine with naphthalene at position 3 in ^68^Ga-DOTANOC results in a higher steric hindrance that destabilizes the interaction between the chelator and the protein, letting the ^68^Ga-DOTA fluctuate more than that for the other compounds (see Figure S3). Conversely, the difference between ^68^Ga-DOTATOC and ^68^Ga-DOTATATE, which share the tyrosine residue at position 3, should be searched in the terminal residue T-ol8/T8. Indeed, the presence of a carboxylic negative charge on the T8 of ^68^Ga-DOTATATE allows the peptide to interact with the basic Arg190, located at ECL2, for 59% of the simulation time (Figures 2B and 3), while this interaction was found neither in ^68^Ga-DOTATOC nor in ^68^Ga-DOTANOC. At the same time, this polar interaction appears also to stabilize a second one between ^68^Ga-DOTA and Arg184 (belonging to ECL2 as well) that in ^68^Ga-DOTATATE was found in 64% of the simulation time, compared to 36 and 19% of ^68^Ga-DOTATOC and ^68^Ga-DOTANOC, respectively. Interestingly, previous studies reported the interaction between Arg184 and somatostatin^20^ and the Arg184Ala mutation was found to decrease the potency of somatostatin, but not that of octreotide.^22^ This last finding supports the crucial role of the deprotonated C-terminus (T8) in the interaction with the receptor. This can possibly explain also the higher selectivity of ^68^Ga-DOTATATE toward the isoform 2, which is characterized by the presence of two arginine residues at the ECL2 (Arg184 and Arg190), whereas ^68^Ga-DOTATOC binds the isoform 5 (belonging to the same sub-class SRIF1) that features on the ECL2 an acid residue (Glu182) instead of a basic one.^20,29^ Further simulations of this class of radiopharmaceuticals interacting with both SSTR2 and SSTR5 are needed to confirm this hypothesis.

The small differences in the peptide structure reflect not only on single-protein residue interaction but also on the overall dynamics of the receptor, especially of the very mobile ECL2. This loop is known to play a key role in the interaction with ligands^30,31^ and it is characterized by opening and closing movements.^20,25^ For this reason, we computed the percentage of MD frames in which the loop was found closed, according to the threshold values established in our previous work.^25^ These thresholds refer to geometric parameters, namely, distances and angles, characterizing the movements of this loop. As a result, ECL2 was closed in about 50, 7, and 18% of the simulation time in ^68^Ga-DOTATOC/TATE/NOC, respectively (Figure S8). The marked differences can trace back to the characteristic behavior of DOTA and T-ol8/T8 moieties in the three compounds described above: in ^68^Ga-DOTATOC, the ^68^Ga-DOTA portion stably interacts with Tyr205^5.35^ (thanks also to the presence of residue Y3 of the peptide), moving this group away from ECL2, and allowing its closure. In ^68^Ga-DOTATATE the interaction with Tyr205^5.35^ is still present, but the ^68^Ga-DOTA moiety also strongly interacts with Arg184, mediated by the T8-Arg190 interaction. Since both arginine residues are located at the ECL2, their involvement in the interaction with the ligand very likely impairs its closure (see below). Differently from the other two compounds, in ^68^Ga-DOTANOC the chelator loosely interacts with Tyr205^5.35^, leading to a higher oscillation that prevents the closure of ECL2.

### Substitution of ^68^Ga^3+^ with ^64^Cu^2+^ Affects the Persistence of Ligand/SSTR2 Interactions

After assessing the role of the peptide moiety, we focused on the influence of the radionuclides by comparing ^64^Cu- and ^68^Ga-DOTATATE. Both gallium and copper ions are hexa-coordinated when in complex with DOTA (by four nitrogen and two oxygen atoms), showing a pseudo-octahedral geometry.^8^ Due to the intrinsic properties of the two radionuclides (*e.g.*, electric charge, van der Waals radius, Jahn–Teller distortion^8^) the coordination geometries differ, showing a more elongated one in ^64^Cu^2+^, compared to ^68^Ga^3+^ (Table S2). Keeping in mind the limitations associated with classical/force field-based MD simulations when describing such challenging types of atoms,^32^ these differences reflected in the conformation assumed by the DOTA group during the MD simulations, where the free/noncoordinating carboxylic acid group of the chelator faces outward when complexed with ^68^Ga^3+^, while it mostly faces inward when complexed with ^64^Cu^2+^ (Figure S9).

**Figure 4:**
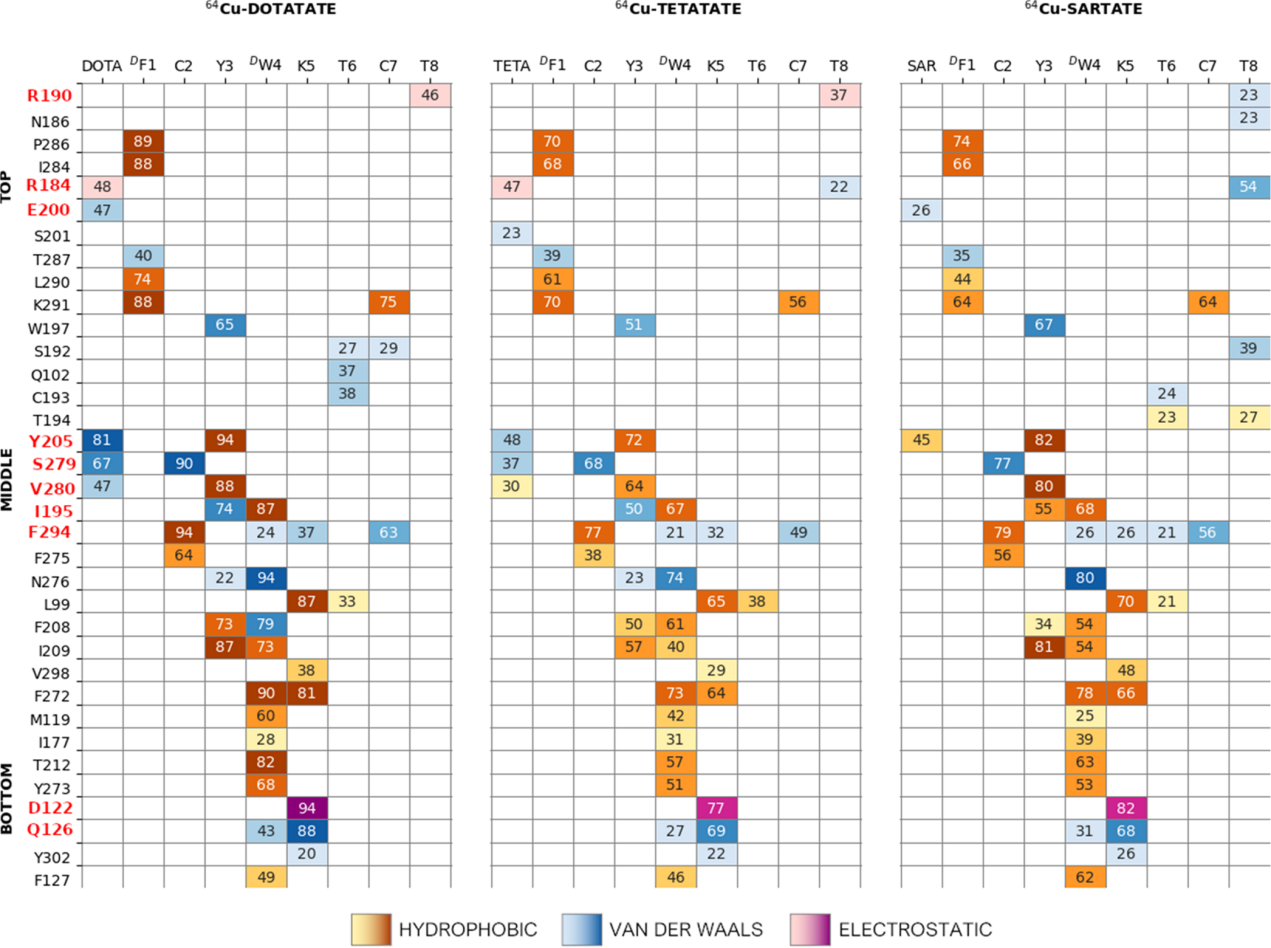
Interaction fingerprint analysis. The values inside the cells report the persistence of the interaction (%) between SSTR2 and ^64^Cu-DOTATATE (left), ^64^Cu-TETATATE (middle), and ^64^Cu-SARTATE (right). For details of the representation code, see the caption of Figure 3. The full fingerprint heatmap is reported in the Supporting Information (Figure S7D–F).

Focusing on the whole ligands, inspection of the dynamical behavior of ^64^Cu-DOTATATE and ^68^Ga-DOTATATE reveals that these compounds share overall the same pattern of interactions with SSTR2. The only relevant differences are found for residue T8 that interacts with Arg184 and Ser192 in ^68^Ga-DOTATATE, and for residue Y3 that interacts with Asn276^6.55^ in ^64^Cu-DOTATATE (Figures 3 and 4). However, these differences do not significantly affect the dynamics of ECL2 (which was found to be closed in the 4 and 7% of the simulation time for ^64^Cu-DOTATATE and ^68^Ga-DOTATATE, respectively) (Figure S8).

### Changes of the Chelator Moiety Influence the Interactions at the Peptide C-Terminal

In the third part of this work, we considered the same radionuclide (^64^Cu^2+^) and the same peptide (TATE) while considering three different chelators (DOTA, TETA, SAR). TETA differs from DOTA just for six atoms (60 vs 54 atoms, respectively), and both coordinate the copper ion through four nitrogen and two oxygen atoms (from the carboxylic groups). Differently from DOTA, in TETA the carboxylic groups are located one above, and one below the plane formed by the nitrogen atoms, conferring a slightly higher steric hindrance (average Connolly surface area^33^ computed on the MD trajectories: 307 ± 3 Å^2^ vs 329 ± 2 Å^2^, respectively). SAR has the lowest surface area (299 ± 2.0 Å^2^) but, differently from the other chelators, it is associated with a butanediamide linker that increases its effective steric hindrance (405 ± 4 Å^2^) as well as its flexibility (Figure S3F). Besides the presence of a linker, another important difference between SAR and the other two chelators is the absence of negatively charged groups (*i.e.*, carboxylic acid moieties), as the chelator coordinates the copper ion through its six nitrogen atoms.

**Figure 5:**
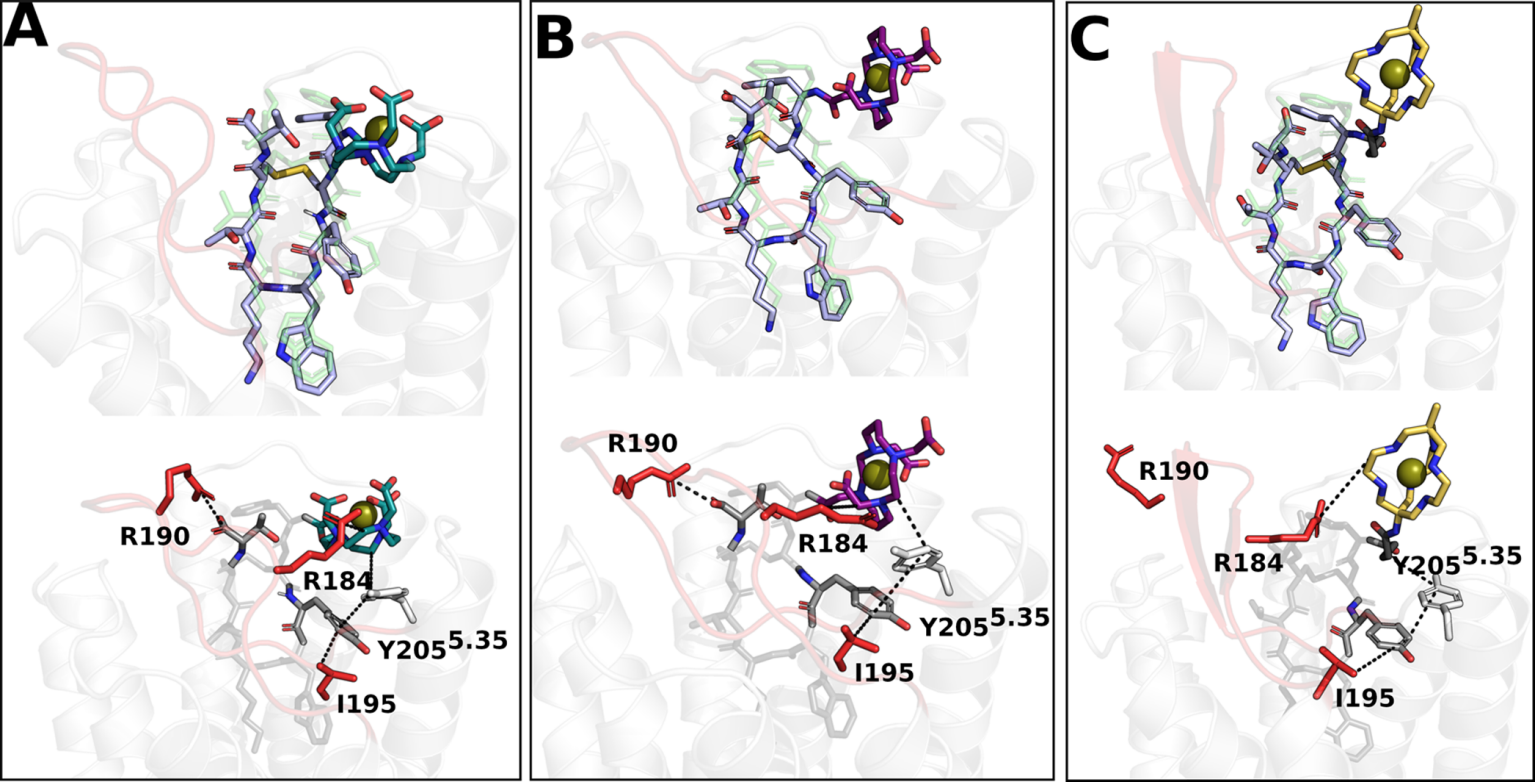
Representatives of the most populated conformational cluster extracted from MD trajectories for (A) ^64^Cu-DOTATATE, (B) ^64^Cu-TETATATE, and (C) ^64^Cu-SARTATE. The corresponding cluster populations are 64.5, 90.8, and 80.3%, respectively. ECL2 is colored in red, the copper ion is represented as a green sphere, the receptor is represented as a transparent white cartoon, the peptide TATE is colored in lilac, the chelators DOTA in dark cyan, TETA in purple, SAR in gold, and its linker in dark gray. The top panels report the superimposition with octreotide (green transparent sticks) taken from PDB7T11. In the bottom panels, the main interactions are shown as black dotted lines.

Focusing on the MD simulations, Figure 5 shows the representatives of the most populated cluster of the ^64^Cu-DOTA/TETA/SAR-TATE compounds. Consistently with what has been reported above for ^68^Ga-based systems, also in this case, we found that all compounds interact with Phe294^7.35^ and Ser279^6.58^ via their disulfide bridge and with Tyr205^5.35^, Ile195, and Val280^6.59^ through Y3 and the chelator moiety (Figure 5). Noteworthy, in contrast to the other radiopharmaceuticals, in ^64^Cu-SARTATE residue Tyr205^5.35^ interacts with the linker portion and not with the chelator (SAR) that remains thus free to oscillate during the MD trajectories.

Combining the clustering of MD trajectories with the interaction fingerprint analysis, we found that DOTA in ^64^Cu-DOTATATE interacts with SSTR2 with the overall higher persistence compared to the other two chelators (TETA and SAR) (Figure 4). As expected, this suggests that the presence of a hindering chelator destabilizes the interaction between the peptide and SSTR2; nonetheless, the improved stability of copper inside such chelator is known to yield high-quality images.^34^

Interestingly, although the TATE peptide was common to all Cu-labeled compounds, the change of the chelator affected the interactions involving the C-terminus and the residues located at the ECL2. In detail, ^64^Cu-DOTA/TETA-TATE interacted with Arg190 through the terminal T8, while the chelator moieties were involved with Arg184. Conversely, in ^64^Cu-SARTATE, the terminal T8 was found to interact mainly with Arg184 (54% of the simulation time) and to a lesser extent with Arg190 (23%), whereas the SAR portion interacted poorly with the ECL2 (only 26% with Glu200) compared to the other chelators (Figures 4 and 5). This different behavior can be traced back to the total +2 net charge of ^64^Cu-SAR (compared to −1 of ^64^Cu-DOTA/TETA) that, despite the presence of a negative C-terminus, penalizes the interactions with basic residues.

Interestingly, when simulating SSTR2 in complex with ^64^Cu-DOTA/TETA-TATE, the ECL2 was found to be closed in 2.7 and 3.5% of the simulation time, respectively, which is consistent with what was registered for ^68^Ga-DOTATATE (6.6%). On the contrary, in ^64^Cu-SARTATE, the ECL2 was able to close upon the binding pocket in 19.1% of the simulation time, similarly to ^68^Ga-DOTANOC (18.1%) (Figure S8). As mentioned above, both ^64^Cu-DOTA/TETA-TATE were able to strongly interact with both Arg184 and Arg190 (such as ^68^Ga-DOTATATE), while ^64^Cu-SARTATE interacted only with Arg184 (like ^68^Ga-DOTATOC/NOC). Therefore, these results suggest that the closure of the ECL2 loop is mostly impaired by the presence of strong polar interactions with the ligands, but also by the high fluctuations of the chelator.

## Conclusions

In this study, we investigated the interaction of six radiopharmaceuticals with SSTR2, a key drug target for neuroendocrine tumors. We predicted the binding modes of these compounds and rationalized the role of the three different moieties characterizing this class (*i.e*., radionuclide-chelator-peptide). Starting from the experimental structure of the receptor in complex with the somatostatin analogous octreotide, we generated the protein–ligand complexes that underwent to overall 15 μs of MD simulation time each. The analysis of the MD trajectories revealed that the substitution of the radionuclide (^68^Ga^3+^ with ^64^Cu^2+^) did not influence the dynamics and the main interactions established by the ligand, while the pattern of interaction of the C-terminus is strongly affected by changes of the chelator moiety (DOTA, TETA, SAR). The radionuclide-chelator portion is stabilized by cross-interactions between Tyr205^5.35^, Ile195, and the third residue of the peptide (Y3 for TOC and TATE, Nal3 for NOC). Furthermore, we found that upon small changes in the peptide structure (at the C-terminal T8/T-ol8 and at the third residue Y3/Nal3), the dynamics of both the chelator portion and SSTR2 strongly differ, possibly paving the way to a molecular rationalization of the differences in SSTR isoform selectivity.

The detailed molecular-level analysis presented in this study and the overall computational platform can be extended to other radiopharmaceuticals of this class, thus contributing to the rational design of new potent ligands targeting SSTR2.

## Computational Details

### System Setup

The starting 3D structure of SSTR2 was retrieved from PDB ID7T11,^20^ in which the receptor was solved in complex with the synthetic agonist octreotide and the G-protein. Missing atoms were added by structure refinement using Modeller10.2.^35^ Given the close similarity between the peptide portion of the six radiopharmaceuticals and octreotide, it was reasonable to assume as the initial position of the ligands in the binding pocket those obtained by direct superimposition between the peptide portion and octreotide. The stability of the initial binding modes was thoroughly tested by monitoring the MD trajectories through analysis of RMSD/F values (see below). To reduce the computational cost, we did not include the G-protein in the structures. The ionization state of the residue side chains, the tautomeric states of histidine residues, and the Asn/Gln flipping were checked by the H++ server.^36^ The CHARMM-GUI server^37^ was used to embed the protein into a double layer of phosphatidylcholine (POPC, 70%) and cholesterol (30%).^38^ The system was inserted in an OPC water box^39^ and neutralized by adding K^+^ and Cl^–^ ions, reaching a 0.15 M concentration. The AmberTools20 software^40^ was used to assign the force field lipid17 to POPC and cholesterol^41^ and ff19SB to the protein.^42^

The peptide portion of the ligands was obtained by manually changing the experimental structure of octreotide, solved in complex with SSTR2 (PDB ID7T11^20^). The chelator structures were retrieved from the Cambridge Structural Database,^43^ choosing those entries solved in complex with a radionuclide (DOTA ID 1136299,^44^ TETA ID 624742,^45^ SAR ID 915824^34^). Finally, the chelator portions carrying the radionuclide were manually bound to the peptide N-terminal. For the generation of the ligand force field parameters, we combined two approaches: (1) one for the peptide and (2) one for the chelator-radionuclide portion. (1) The force field ff19SB was assigned to the peptide, and nonstandard residues (*i.e.*, d-phenylalanine, naphthalene, d-tryptophan, threoninol) were parametrized as described previously.^25^ (2) Given the peculiarity of the metal coordination bond involving ^68^Ga^3+^ and ^64^Cu^2+^ and their challenging parametrization, we used the Metal Center Parameter Builder (MCPB.py) procedure^46^ implemented in Amber20. In detail, the 3D structures of the chelator-radionuclide and the first residue of the ligands (^D^F1), obtained as described above, underwent quantum mechanics (QM) calculations at the Density Functional Theory (DFT) level^47^ with the B3LYP functional using the Gaussian16 package (Revision A.03).^48^ We performed geometry optimization on ^68^Ga-DOTA using different basis sets in order to identify the best one for our system (Table S2). We compared the coordinaion distances between the experimental and QM optimized structure, and we computed the mean absolute error results (MAE). We observed that a large basis set does not lead to big differences in geometry optimization. Therefore, we employed the hybrid B3LYP functional,^49^ in conjunction with the split-valence 6-31G(d,p) Gaussian basis set^50^ to save computational time. For each compound, the ground-state structure was optimized, and then a full vibrational analysis was performed. In the case of ^64^Cu-SAR, solvation effects were calculated using the integral equation formalism of the Polarized Continuum Model (IEF-PCM),^51^ with water as the solvent^52^ to avoid the collapse of the chelator. In all cases, the DFT-based structural parameters are in good agreement with the available experimental data (Table S2). The vibrational analysis results were used by MCPB.py to generate the bonded terms of the force fields. Then, on the optimized geometry we performed B3LYP/6-31G(d,p) single-point energy calculations to generate the atomic partial charges fitting the molecular electrostatic potential. We used the Merz–Kollman scheme^53^ to construct a grid of points around the molecule under the constraint of reproducing the overall electric dipole moment of the molecule. Atomic partial charges were then generated through the two-step restrained electrostatic potential (RESP) method^54^ implemented in the Antechamber package.^55^ These steps enabled the generation of the force field of the chelator-radionuclide moieties using the General Amber Force Field 2 (GAFF2).^56^

### MD Simulations

Each system underwent an energy minimization combining the steepest-descent and the conjugated gradient algorithms (2500 steps each) and applying positional restraints on the protein–ligand complex (10.0 kcal mol^–1^ Å^–2^) and on cholesterol and phosphate groups of phosphatidylcholine molecules (2.5 kcal mol^–1^ Å^–2^).

NVT and NPT equilibrations followed minimization, in which the positional restraints were incrementally reduced. The NVT equilibration was divided into two steps: first 125 ps with the same positional restraints of the minimization, then further 125 ps decreasing the restraint strength to 5.0 kcal mol^–1^ Å^–2^ for the protein and the ligands, and keeping 2.5 kcal mol^–1^ Å^–2^ for cholesterol and the phosphate groups of phosphatidylcholine molecules (overall NVT equilibration time = 250 ps). The following NPT equilibration was divided into four steps: (1) 125 ps using positional restraints of 2.5 kcal mol^–1^ Å^–2^ for the protein–ligand, and 1.0 kcal mol^–1^ Å^–2^ for the membrane components, (2) 500 ps using 2.5 kcal mol^–1^ Å^–2^ for protein–ligand and 1.0 kcal mol^–1^ Å^–2^ for the membrane, (3) 500 ps using 0.5 kcal mol^–1^ Å^–2^ for the protein–ligand and 0.1 kcal mol^–1^ Å^–2^ for the membrane, and finally (4) 500 ps using 0.1 kcal mol^–1^ Å^–2^ for the protein–ligand and leaving the membrane completely free to move (overall NPT equilibration time = 1.625 ns). We used the Langevin thermostat (1 ps^–1^ as collision frequency, 310 K) and the Berendsen barostat (1 atm), a cutoff of 9 Å, the time step was incremented from 1 to 2 fs with the SHAKE algorithm,^57^ the Particle Mesh Ewald method for long-range electrostatics was employed.^58^ The production runs were carried on for 3 μs, using the NPT ensemble and 4 fs as a time step, adopting the hydrogen mass repartition scheme.^59^ Five replicas were generated for each system, resulting in a 15 μs simulation time. The MD simulations were conducted using the PMEMD module of Amber20,^40^ and the trajectory frames were written every 100 ps.

### MD Trajectory Analyses

MD replicas were first concatenated and the CPPTRAJ software^60^ was used to perform a cluster analysis. A hierarchical algorithm^61^ was used to group all frames into conformational clusters, according to the compound RMSD. Considering all cases, we found that three clusters sample significantly different conformations of the ligands which are, at the same time, reasonably populated (see Table S1). In all cases, the RMSD values of the ligands were computed on all of the heavy atoms, after aligning the backbone of the receptor in the MD trajectories, with respect to the first frame of the production run. Interaction fingerprints were computed using the ProLIF Python library^62^ on all of the frames of the MD trajectories. The numbers of interactions were combined for all replicas and converted into persistence of interactions (%).

## Data Availability

PDB files were downloaded from the RCSB Protein Data Bank (https://www.rcsb.org). AMBER20 was used to perform MD simulations and trajectory analysis (https://ambermd.org/). Fingerprint analysis was performed with ProLIF available at https://github.com/chemosim-lab/ProLIF. General Amber force field parameters for ligands were obtained using MCPB.py (https://ambermd.org/tutorials/advanced/tutorial20/). PyMOL 2.4.0 was used for molecular visualization (https://pymol.org/2/). Marvin ChemAxon 19.24 and GIMP 2.10 were used for the generation of molecular and general graphics (https://docs.chemaxon.com/, https://www.gimp.org/). The input topology (PARM7) and coordinate files (RST7), the input files of QM and MD calculations, together with the raw files of the MD trajectories (XTC), and the conformational clusters representatives (PDB) are freely available at Zenodo (DOI 10.5281/zenodo.7927992).

## Supporting Information Available

SSTR2 domains, RMSD/F, cluster and fingerprint analyses, ECL2 opening and closing movements, and QM geometry optimization results (PDF)

## Abbreviations

NET: neuroendocrine tumors
GPCR: G-protein coupled receptor
SSTR: somatostatin receptor
FDA: Food and Drug Administration
TM: transmembrane helix
ECL: extracellular loop
ICL: intracellular loop
DOTA: 1,4,7,10-tetraazacyclododecane-*N*,*N*′,*N*″,*N*‴-tetraacetic acid
TETA: 1,4,8,11-tetraazacyclotetradecane-1,4,8,11-tetraacetic acid
SAR: 3,6,10,13,16,19-hexazabicyclo[6.6.6]icosane
TOC: tyrosine-3-octreotide
TATE: tyrosine-3-octreotate
NOC: naphthalene-3-octreotide
PET: positron emission tomography
SPECT: single-photon emission computed tomography
RMSD: root-mean-square deviation
RMSF: root-mean-square fluctuation

## References

[ref1] Goetz L. H.; Schork N. J. Personalized Medicine: Motivation, Challenges, and Progress. Fertil. Steril. 2018, 109 (6), 952–963. 10.1016/j.fertnstert.2018.05.006. 29935653 PMC6366451

[ref2] Jackson S. E.; Chester J. D. Personalised Cancer Medicine. Int. J. Cancer 2015, 137 (2), 262–266. 10.1002/ijc.28940. 24789362

[ref3] Kręcisz P.; Czarnecka K.; Kroíicki L.; Bieta Mikiciuk-Olasik E.; Szymański P. S. Radiolabeled Peptides and Antibodies in Medicine. Bioconjugate Chem. 2021, 32 (1), 25–42. 10.1021/acs.bioconjchem.0c00617. PMC7872318 33325685

[ref4] Lengacher R.; Marlin A.; Śmiłowicz D.; Boros E. Medicinal Inorganic Chemistry – Challenges, Opportunities and Guidelines to Develop the next Generation of Radioactive, Photoactivated and Active Site Inhibiting Metal-Based Medicines. Chem. Soc. Rev. 2022, 51 (18), 7715–7731. 10.1039/D2CS00407K. 35942718

[ref5] Holik H. A.; Ibrahim F. M.; Elaine A. A.; Putra B. D.; Achmad A.; Kartamihardja A. H. S. The Chemical Scaffold of Theranostic Radiopharmaceuticals: Radionuclide, Bifunctional Chelator, and Pharmacokinetics Modifying Linker. Molecules 2022, 27 (10), 3062 10.3390/molecules27103062. 35630536 PMC9143622

[ref6] Eychenne R.; Bouvry C.; Bourgeois M.; Loyer P.; Benoist E.; Lepareur N. Overview of Radiolabeled Somatostatin Analogs for Cancer Imaging and Therapy. Molecules 2020, 25 (17), 4012 10.3390/molecules25174012. 32887456 PMC7504749

[ref7] Sheikhbahaei S.; Sadaghiani M. S.; Rowe S. P.; Solnes L. B. Neuroendocrine Tumor Theranostics: An Update and Emerging Applications in Clinical Practice. Am. J. Roentgenol. 2021, 217 (2), 495–506. 10.2214/AJR.20.23349. 34076455

[ref8] Wadas T. J.; Wong E. H.; Weisman G. R.; Anderson C. J. Coordinating Radiometals of Copper, Gallium, Indium, Yttrium, and Zirconium for PET and SPECT Imaging of Disease. Chem. Rev. 2010, 110 (5), 2858–2902. 10.1021/cr900325h. 20415480 PMC2874951

[ref9] Anderson C. J.; Pajeau T. S.; Edwards W. B.; Sherman E. L. C.; Rogers B. E.; Welch M. J. In Vitro and In Vivo Evaluation of Copper-64-Octreotide Conjugates. J. Nucl. Med. 1995, 36 (12), 2315–2325. 8523125

[ref10] Vahidfar N.; Farzanehfar S.; Abbasi M.; Mirzaei S.; Delpassand E. S.; Abbaspour F.; Salehi Y.; Biersack H. J.; Ahmadzadehfar H. Diagnostic Value of Radiolabelled Somatostatin Analogues for Neuroendocrine Tumour Diagnosis: The Benefits and Drawbacks of [64Cu]Cu-DOTA-TOC. Cancers 2022, 14 (8), 1914 10.3390/cancers14081914. 35454822 PMC9027354

[ref11] Oronsky B.; Ma P. C.; Morgensztern D.; Carter C. A. Nothing But NET: A Review of Neuroendocrine Tumors and Carcinomas. Neoplasia 2017, 19 (12), 991–1002. 10.1016/j.neo.2017.09.002. 29091800 PMC5678742

[ref12] Stueven A. K.; Kayser A.; Wetz C.; Amthauer H.; Wree A.; Tacke F.; Wiedenmann B.; Roderburg C.; Jann H. Somatostatin Analogues in the Treatment of Neuroendocrine Tumors: Past, Present and Future. Int. J. Mol. Sci. 2019, 20 (12), 3049 10.3390/ijms20123049. 31234481 PMC6627451

[ref13] Zhao W.; Han S.; Qiu N.; Feng W.; Lu M.; Zhang W.; Wang M.; Zhou Q.; Chen S.; Xu W.; Du J.; Chu X.; Yi C.; Dai A.; Hu L.; Shen M. Y.; Sun Y.; Zhang Q.; Ma Y.; Zhong W.; Yang D.; Wang M. W.; Wu B.; Zhao Q. Structural Insights into Ligand Recognition and Selectivity of Somatostatin Receptors. Cell Res. 2022, 32, 761–772. 10.1038/s41422-022-00679-x. 35739238 PMC9343605

[ref14] Günther T.; Tulipano G.; Dournaud P.; Bousquet C.; Csaba Z.; Kreienkamp H. J.; Lupp A.; Korbonits M.; Castaño J. P.; Wester H. J.; Culler M.; Melmed S.; Schulz S. International Union of Basic and Clinical Pharmacology. CV. Somatostatin Receptors: Structure, Function, Ligands, and New Nomenclature. Pharmacol. Rev. 2018, 70 (4), 763–835. 10.1124/pr.117.015388. 30232095 PMC6148080

[ref15] Bo Q.; Yang F.; Li Y.; Meng X.; Zhang H.; Zhou Y.; Ling S.; Sun D.; Lv P.; Liu L.; Shi P.; Tian C. Structural Insights into the Activation of Somatostatin Receptor 2 by Cyclic SST Analogues. Cell Discovery 2022, 8, 47 10.1038/s41421-022-00405-2. 35595746 PMC9122944

[ref16] Hu Y.; Ye Z.; Wang F.; Qin Y.; Xu X.; Yu X.; Ji S. Role of Somatostatin Receptor in Pancreatic Neuroendocrine Tumor Development, Diagnosis, and Therapy. Front. Endocrinol. 2021, 12, 679000 10.3389/fendo.2021.679000. PMC8170475 34093445

[ref17] Harris P. E.; Zhernosekov K. The Evolution of PRRT for the Treatment of Neuroendocrine Tumors; What Comes Next?. Front. Endocrinol. 2022, 13, 941832 10.3389/fendo.2022.941832. PMC9659917 36387893

[ref18] Menda Y.; Madsen M. T.; O’Dorisio T. M.; Sunderland J. J.; Watkins G. L.; Dillon J. S.; Mott S. L.; Schultz M. K.; Zamba G. K. D.; Bushnell D. L.; O’Dorisio M. S. 90Y-DOTATOC Dosimetry-Based Personalized Peptide Receptor Radionuclide Therapy. J. Nucl. Med. 2018, 59 (11), 1692–1698. 10.2967/jnumed.117.202903. 29523629 PMC6225542

[ref19] del Olmo-García M. I.; Prado-Wohlwend S.; Bello P.; Segura A.; Merino-Torres J. F. Peptide Receptor Radionuclide Therapy with [177Lu]Lu-DOTA-TATE in Patients with Advanced GEP NENS: Present and Future Directions. Cancers 2022, 14 (3), 584 10.3390/cancers14030584. 35158852 PMC8833790

[ref20] Robertson M. J.; Meyerowitz J. G.; Panova O.; Borrelli K.; Skiniotis G. Plasticity in Ligand Recognition at Somatostatin Receptors. Nat. Struct. Mol. Biol. 2022, 29, 210–217. 10.1038/s41594-022-00727-5. 35210615 PMC11073612

[ref21] Chen L. N.; Wang W. W.; Dong Y. J.; Shen D. D.; Guo J.; Yu X.; Qin J.; Ji S. Y.; Zhang H.; Shen Q.; He Q.; Yang B.; Zhang Y.; Li Q.; Mao C. Structures of the Endogenous Peptide- and Selective Non-Peptide Agonist-Bound SSTR2 Signaling Complexes. Cell Res. 2022, 32, 785–788. 10.1038/s41422-022-00669-z. 35578016 PMC9343650

[ref22] Chen S.; Teng X.; Zheng S. Molecular Basis for the Selective G Protein Signaling of Somatostatin Receptors. Nat. Chem. Biol. 2023, 19, 133–140. 10.1038/s41589-022-01130-3. 36138141

[ref23] Zhao J.; Fu H.; Yu J.; Hong W.; Tian X.; Qi J.; Sun S.; Zhao C.; Wu C.; Xu Z.; Cheng L.; Chai R.; Yan W.; Wei X.; Shao Z. Prospect of Acromegaly Therapy: Molecular Mechanism of Clinical Drugs Octreotide and Paltusotine. Nat. Commun. 2023, 14, 962 10.1038/s41467-023-36673-z. 36810324 PMC9944328

[ref24] Salas-Estrada L.; Fiorillo B.; Filizola M. Metadynamics Simulations Leveraged by Statistical Analyses and Artificial Intelligence-Based Tools to Inform the Discovery of G Protein-Coupled Receptor Ligands. Front. Endocrinol. 2022, 13, 1099715 10.3389/fendo.2022.1099715. PMC9816996 36619585

[ref25] Gervasoni S.; Guccione C.; Fanti V.; Bosin A.; Cappellini G.; Golosio B.; Ruggerone P.; Malloci G. Molecular Simulations of SSTR2 Dynamics and Interaction with Ligands. Sci. Rep. 2023, 13, 4768 10.1038/s41598-023-31823-1. 36959237 PMC10036620

[ref26] Ishida A.; Tajima Y.; Okabe Y.; Matsushita T.; Sekiguchi T.; Imaide S.; Nomura Y.; Tanaka M.; Nojima S.; Yoshida A.; Iyoda Y.; Aoki S.; Nishio T.; Komagata T.; Iwaki M.; Shono T.; Naganawa A.; Imagawa A. Discovery and SAR Studies of Orally Active Somatostatin Receptor Subtype-2 (SSTR2) Agonists for the Treatment of Acromegaly. ACS Chem. Neurosci. 2020, 11 (10), 1482–1494. 10.1021/acschemneuro.0c00124. 32315148

[ref27] Isberg V.; De Graaf C.; Bortolato A.; Cherezov V.; Katritch V.; Marshall F. H.; Mordalski S.; Pin J. P.; Stevens R. C.; Vriend G.; Gloriam D. E. Generic GPCR Residue Numbers – Aligning Topology Maps While Minding the Gaps. Trends Pharmacol. Sci. 2015, 36 (1), 22–31. 10.1016/j.tips.2014.11.001. 25541108 PMC4408928

[ref28] Heo Y.; Yoon E.; Jeon Y. E.; Yun J. H.; Ishimoto N.; Woo H.; Park S. Y.; Song J. J.; Lee W. Cryo-EM Structure of the Human Somatostatin Receptor 2 Complex with Its Agonist Somatostatin Delineates the Ligand-Binding Specificity. eLife 2022, 36 (1), 22–31. 10.7554/ELIFE.76823. PMC9054131 35446253

[ref29] Barca C.; Griessinger C. M.; Faust A.; Depke D.; Essler M.; Windhorst A. D.; Devoogdt N.; Brindle K. M.; Schäfers M.; Zinnhardt B.; Jacobs A. H. Expanding Theranostic Radiopharmaceuticals for Tumor Diagnosis and Therapy. Pharmaceuticals 2022, 15 (1), 13 10.3390/ph15010013. PMC8780589 35056071

[ref30] Nicoli A.; Dunkel A.; Giorgino T.; De Graaf C.; Di Pizio A. Classification Model for the Second Extracellular Loop of Class A GPCRs. J. Chem. Inf. Model. 2022, 62 (3), 511–522. 10.1021/acs.jcim.1c01056. 35113559

[ref31] Woolley M. J.; Conner A. C. Understanding the Common Themes and Diverse Roles of the Second Extracellular Loop (ECL2) of the GPCR Super-Family. Mol. Cell. Endocrinol. 2017, 449, 3–11. 10.1016/j.mce.2016.11.023. 27899324

[ref32] Li P.; Merz K. M. Metal Ion Modeling Using Classical Mechanics. Chem. Rev. 2017, 117 (3), 1564–1686. 10.1021/acs.chemrev.6b00440. 28045509 PMC5312828

[ref33] Connolly M. L. Analytical Molecular Surface Calculation. J. Appl. Crystallogr. 1983, 16, 548–558. 10.1107/S0021889883010985.

[ref34] Paterson B. M.; Roselt P.; Denoyer D.; Cullinane C.; Binns D.; Noonan W.; Jeffery C. M.; Price R. I.; White J. M.; Hicks R. J.; Donnelly P. S. PET Imaging of Tumours with a 64Cu Labeled Macrobicyclic Cage Amine Ligand Tethered to Tyr3-Octreotate. Dalton Trans. 2014, 43, 1386–1396. 10.1039/C3DT52647J. 24202174

[ref35] Webb B.; Sali A. Comparative Protein Structure Modeling Using MODELLER. In Current Protocols in Bioinformatics; Wiley, 2016; Vol. 54, pp 5–6. 10.1002/cpbi.3 PMC5031415 27322406

[ref36] Gordon J. C.; Myers J. B.; Folta T.; Shoja V.; Heath L. S.; Onufriev A. H++: A Server for Estimating p Ka s and Adding Missing Hydrogens to Macromolecules. Nucleic Acids Res. 2005, 33 (Suppl_2), W368–W371. 10.1093/NAR/GKI464. 15980491 PMC1160225

[ref37] Jo S.; Kim T.; Iyer V. G.; Im W. CHARMM-GUI: A Web-Based Graphical User Interface for CHARMM. J. Comput. Chem. 2008, 29, 1859–1865. 10.1002/jcc.20945. 18351591

[ref38] Saeedimasine M.; Montanino A.; Kleiven S.; Villa A. Role of Lipid Composition on the Structural and Mechanical Features of Axonal Membranes: A Molecular Simulation Study. Sci. Rep. 2019, 9, 8000 10.1038/s41598-019-44318-9. 31142762 PMC6541598

[ref39] Izadi S.; Anandakrishnan R.; Onufriev A. V. Building Water Models: A Different Approach. J. Phys. Chem. Lett. 2014, 5 (21), 3863–3871. 10.1021/jz501780a. 25400877 PMC4226301

[ref40] Case D. A.; Aktulga H. M.; Belfon K.; Ben-Shalom I. Y.; Berryman J. T.; Brozell S. R.; Cerutti D. S.; Cheatham T. E. III; Cisneros G. A.; Cruzeiro V.W.D.; Darden T. A.; Duke R. E.; Giambasu G.; Gilson M. K.; Gohlke H.; Goetz A. W.; Harris R.; Izadi S.; Izmailov S. A.; Amber P. A. K. Amber 2022; University of California: San Francisco, 2022.

[ref41] Gould I. R.; Skjevik A. A.; Dickson C. J.; Madej B. D.; Walker R. C. Lipid17: A Comprehensive AMBER Force Field for the Simulation of Zwitterionic and Anionic Lipids. Manuscript in preparation, 2018.

[ref42] Tian C.; Kasavajhala K.; Belfon K. A. A.; Raguette L.; Huang H.; Migues A. N.; Bickel J.; Wang Y.; Pincay J.; Wu Q.; Simmerling C. Ff19SB: Amino-Acid-Specific Protein Backbone Parameters Trained against Quantum Mechanics Energy Surfaces in Solution. J. Chem. Theory Comput. 2020, 16 (1), 528–552. 10.1021/acs.jctc.9b00591. 31714766 PMC13071887

[ref43] Groom C. R.; Bruno I. J.; Lightfoot M. P.; Ward S. C. The Cambridge Structural Database. Acta Crystallogr., Sect. B: Struct. Sci., Cryst. Eng. Mater. 2016, 72 (2), 171–179. 10.1107/S2052520616003954. PMC4822653 27048719

[ref44] Riesen A.; Zehnder M.; Kaden T. A. Metal Complexes of Macrocyclic Ligands. Part XXIV. Binuclear Complexes with Tetraazamacrocycle-N,N′,N″,N‴-Tetraacetic Acids. Helv. Chim. Acta 1986, 69 (8), 2074–2080. 10.1002/hlca.19860690831.

[ref45] Silversides J. D.; Allan C. C.; Archibald S. J. Copper(II) Cyclam-Based Complexes for Radiopharmaceutical Applications: Synthesis and Structural Analysis. Dalton Trans. 2007, (9), 971–978. 10.1039/b615329a. 17308678 PMC1978068

[ref46] Li P.; Merz K. M. Parameterization of a Dioxygen Binding Metal Site Using the MCPB.Py Program. Methods Mol. Biol. 2021, 2199, 257–275. 10.1007/978-1-0716-0892-0_15. 33125655

[ref47] Kohn W. Electronic Structure of Matter - Wave Functions and Density Functional. Rev. Mod. Phys. 1999, 71 (5), 1253–1266. 10.1103/RevModPhys.71.1253.

[ref48] Frisch M. J.; Trucks G. W.; Schlegel H. B.; Scuseria G. E.; Robb M. A.; Cheeseman J. R.; Scalmani G.; Barone V.; Petersson G. A.; Nakatsuji H.; Li X.; Caricato M.; Marenich A. V.; Bloino J.; Janesko B. G.; Gomperts R.; Mennucci B.; Hratchian H. P.; Ortiz J. V.; Fox D. J. Gaussian 16, revision A.03; Gaussian, Inc.: Wallingford, CT, 2016.

[ref49] Becke A. D. Density-functional Thermochemistry. III. The Role of Exact Exchange. J. Chem. Phys. 1993, 98, 5648 10.1063/1.464913.

[ref50] Pople J. A. Quantum Chemical Models. Angew. Chem., Int. Ed. 1999, 38, 1894–1902. 10.1002/(SICI)1521-3773(19990712)38:13/14<1894::AID-ANIE1894>3.0.CO;2-H. 34182688

[ref51] Tomasi J.; Mennucci B.; Cammi R. Quantum Mechanical Continuum Solvation Models. Chem. Rev. 2005, 105 (8), 2999–3093. 10.1021/cr9904009. 16092826

[ref52] Miertuš S.; Scrocco E.; Tomasi J. Electrostatic Interaction of a Solute with a Continuum. A Direct Utilizaion of AB Initio Molecular Potentials for the Prevision of Solvent Effects. Chem. Phys. 1981, 55 (1), 117–129. 10.1016/0301-0104(81)85090-2.

[ref53] Singh U. C.; Kollman P. A. An Approach to Computing Electrostatic Charges for Molecules. J. Comput. Chem. 1984, 5, 129–145. 10.1002/jcc.540050204.

[ref54] Bayly C. I.; Cieplak P.; Cornell W. D.; Kollman P. A. A Well-Behaved Electrostatic Potential Based Method Using Charge Restraints for Deriving Atomic Charges: The RESP Model. J. Phys. Chem. A 1993, 97 (40), 10269–10280. 10.1021/j100142a004.

[ref55] Wang J.; Wang W.; Kollman P. A.; Case D. A. Automatic Atom Type and Bond Type Perception in Molecular Mechanical Calculations. J. Mol. Graphics Modell. 2006, 25 (2), 247–260. 10.1016/j.jmgm.2005.12.005. 16458552

[ref56] Wang J.; Wolf R. M.; Caldwell J. W.; Kollman P. A.; Case D. A. Development and Testing of a General Amber Force Field. J. Comput. Chem. 2004, 25 (9), 1157–1174. 10.1002/jcc.20035. 15116359

[ref57] Kräutler V.; Van Gunsteren W. F.; Hünenberger P. H. A Fast SHAKE Algorithm to Solve Distance Constraint Equations for Small Molecules in Molecular Dynamics Simulations. J. Comput. Chem. 2001, 22, 501–508. 10.1002/1096-987X.

[ref58] Darden T.; York D.; Pedersen L. Particle Mesh Ewald: An N·log(N) Method for Ewald Sums in Large Systems. J. Chem. Phys. 1993, 98, 10089 10.1063/1.464397.

[ref59] Jung J.; Kasahara K.; Kobayashi C.; Oshima H.; Mori T.; Sugita Y. Optimized Hydrogen Mass Repartitioning Scheme Combined with Accurate Temperature/Pressure Evaluations for Thermodynamic and Kinetic Properties of Biological Systems. J. Chem. Theory Comput. 2021, 17 (8), 5312–5321. 10.1021/acs.jctc.1c00185. 34278793

[ref60] Roe D. R.; Cheatham T. E. PTRAJ and CPPTRAJ: Software for Processing and Analysis of Molecular Dynamics Trajectory Data. J. Chem. Theory Comput. 2013, 9 (7), 3084–3095. 10.1021/ct400341p. 26583988

[ref61] Shao J.; Tanner S. W.; Thompson N.; Cheatham T. E. Clustering Molecular Dynamics Trajectories: 1. Characterizing the Performance of Different Clustering Algorithms. J. Chem. Theory Comput. 2007, 3 (6), 2312–2334. 10.1021/ct700119m. 26636222

[ref62] Bouysset C.; Fiorucci S. ProLIF: A Library to Encode Molecular Interactions as Fingerprints. J. Cheminf. 2021, 13, 72 10.1186/s13321-021-00548-6. PMC8466659 34563256

