# Supplementary material for: Interaction of Radiopharmaceuticals with Somatostatin Receptor 2 Revealed by Molecular Dynamics Simulations
Source: J Chem Inf Model. 2023 Jul 19;63(15):4924–33. doi: 10.1021/acs.jcim.3c00712 (PMC10428218; doi:10.1021/acs.jcim.3c00712)
Supplement: Supplementary file 1 — ci3c00712_si_001.pdf [file ci3c00712_si_001.pdf]

Supporting Information

INTERACTION OF RADIOPHARMACEUTICALS WITH SOMATOSTATIN RECEPTOR 2 REVEALED BY MOLECULAR DYNAMICS SIMULATIONS

Silvia Gervasoni‡*, Işılay Öztürk‡, Camilla Guccione, Andrea Bosin, Paolo Ruggerone and Giuliano Malloci*

University of Cagliari, Department of Physics, Monserrato (Cagliari), I-09042, Italy

Email: giuliano.malloci@dsf.unica.it, silvia.gervasoni@dsf.unica.it

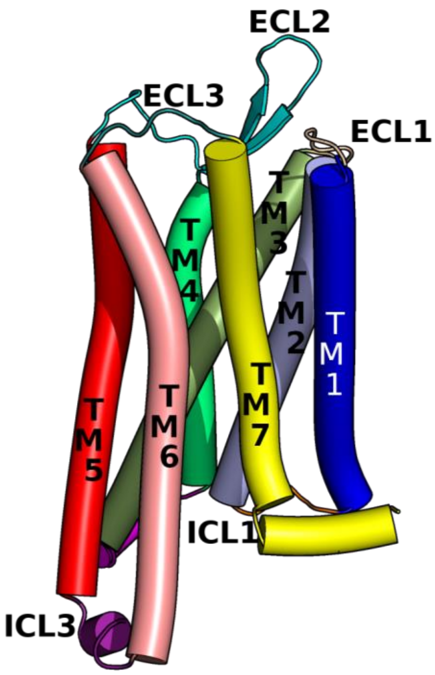

Figure S1. Overview of SSTR2 structure and domains. TM= transmembrane helix, ECL= extracellular loop, ICL= intracellular loop.

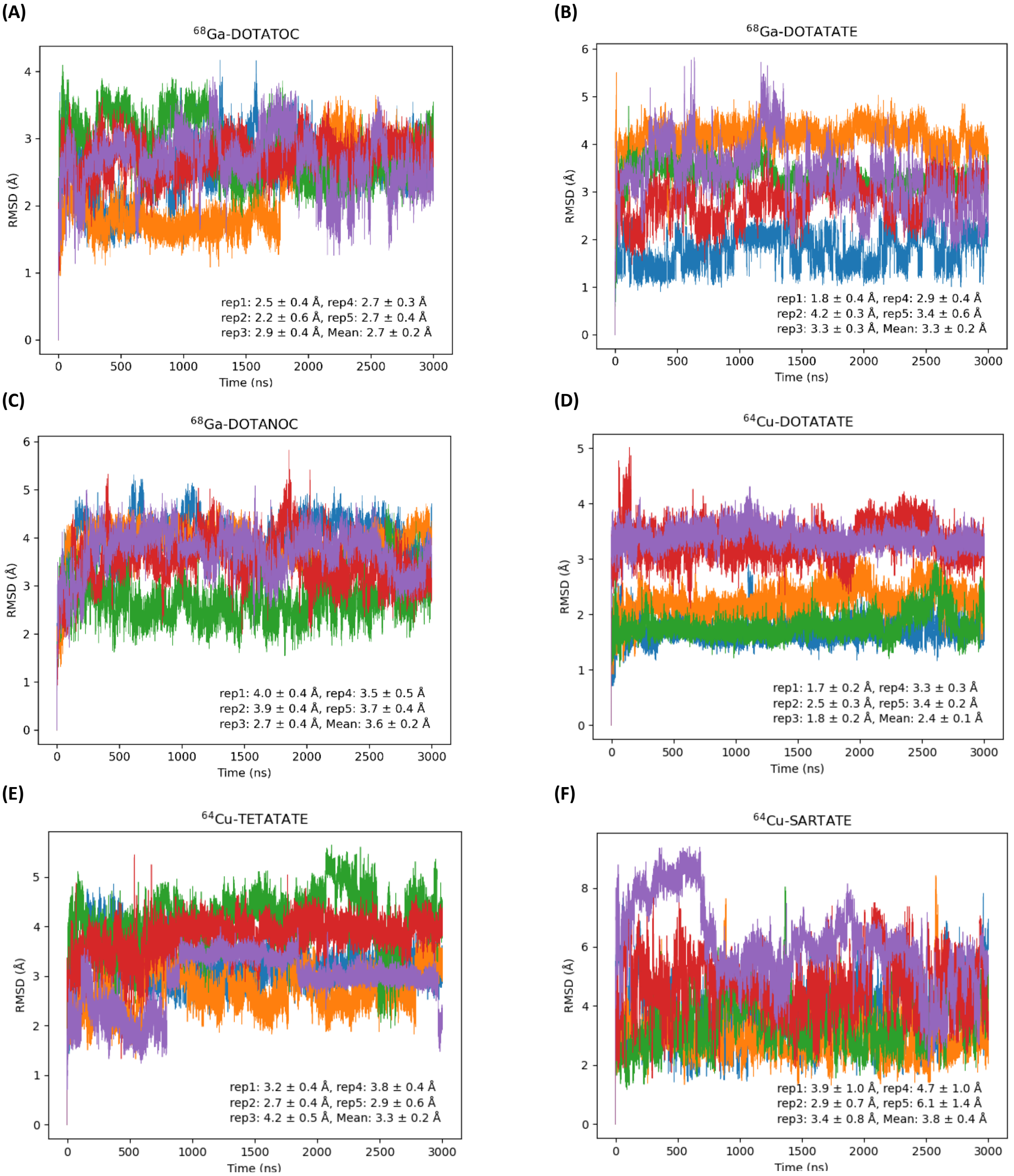

Figure S2. RMSD values (Å) of the ligands, computed on the heavy atoms with respect to the first frame of the MD trajectory. **(A)** ^{68}Ga-DOTATOC, **(B)** ^{68}Ga-DOTATATE, **(C)** ^{68}GA-DOTANOC, **(D)** ^{64}Cu-DOTATATE, **(E)** ^{64}Cu-TETATATE, **(F)** ^{64}Cu-SARTATE. Mean values of every replica and the overall mean values, with the corresponding standard deviations, are also reported.

(A)

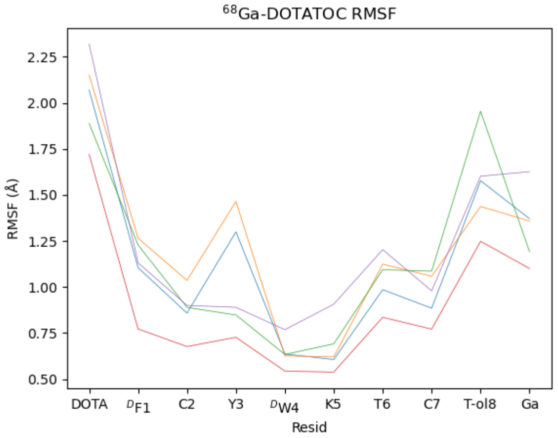

(B)

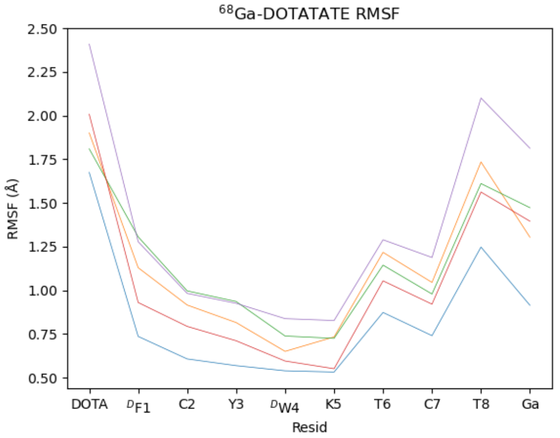

(C)

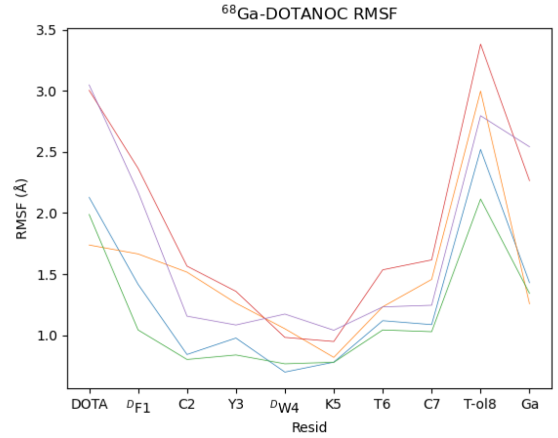

(D)

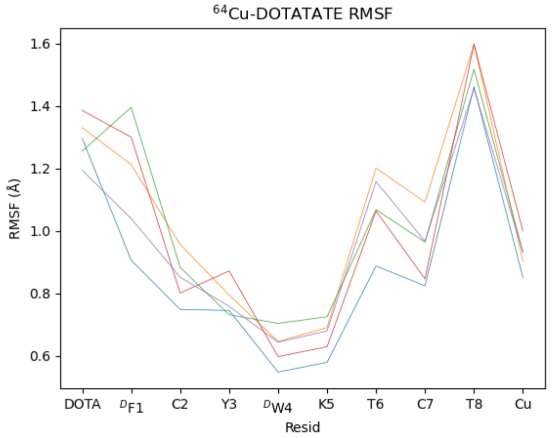

(E)

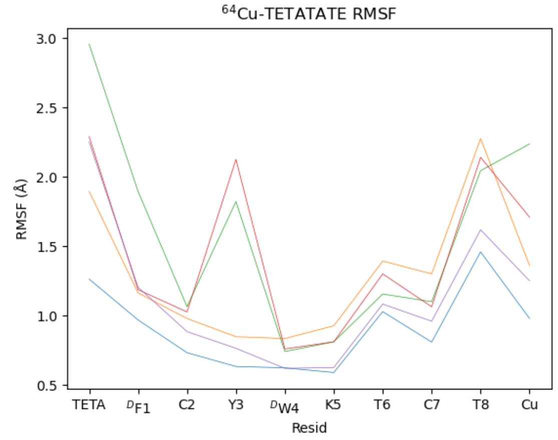

(F)

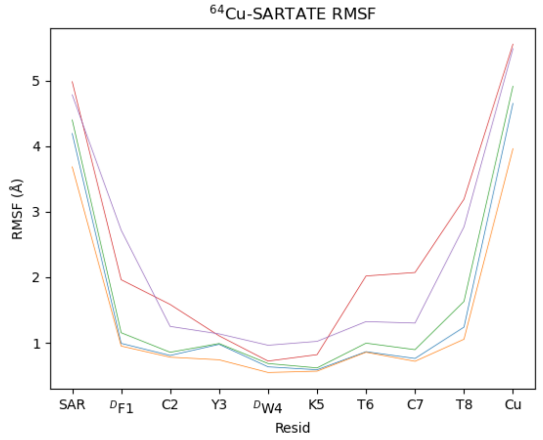

Figure S3. RMSF values (Å) of the ligands, computed on the heavy atoms. **(A)** ^{68}Ga-DOTATOC, **(B)** ^{68}Ga-DOTATATE, **(C)** ^{68}GA-DOTANOC, **(D)** ^{64}Cu-DOTATATE, **(E)** ^{64}Cu-TETATATE, **(F)** ^{64}Cu-SARTATE.

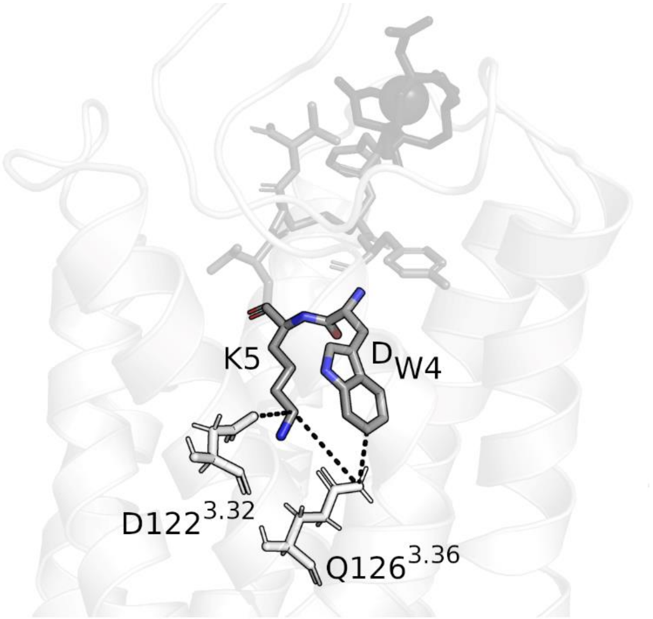

Figure S4. Conserved interactions between ligands and SSTR2, located at the bottom of the binding pocket. ^{64}Ga-DOTATATE is shown as an exemplification.

Table S1. Results of the cluster analysis. The population of each cluster is reported in percentage. The RMSD values (Å) between the cluster representatives, computed on the heavy atoms of the ligands, are also listed.

	**Population (%)**			**RMSD (Å)**		
	**c0**	**c1**	**c2**	**c1 vs c0**	**c2 vs c0**	**c1 vs c2**
**^{68}Ga-DOTATOC**	57.3	28.5	14.2	1.3	1.7	2.3
**^{68}Ga-DOTATATE**	67.3	31.3	1.4	1.4	2.7	3.1
**^{68}Ga-DOTANOC**	78.0	14.8	7.1	3.1	2.8	2.8
**^{64}Cu-DOTATATE**	64.5	35.5	0.1	0.9	2.5	2.4
**^{64}Cu-TETATATE**	90.8	7.6	1.6	2.0	3.7	3.0
**^{64}Cu-SARTATE**	80.3	13.5	6.2	3.0	4.7	3.6

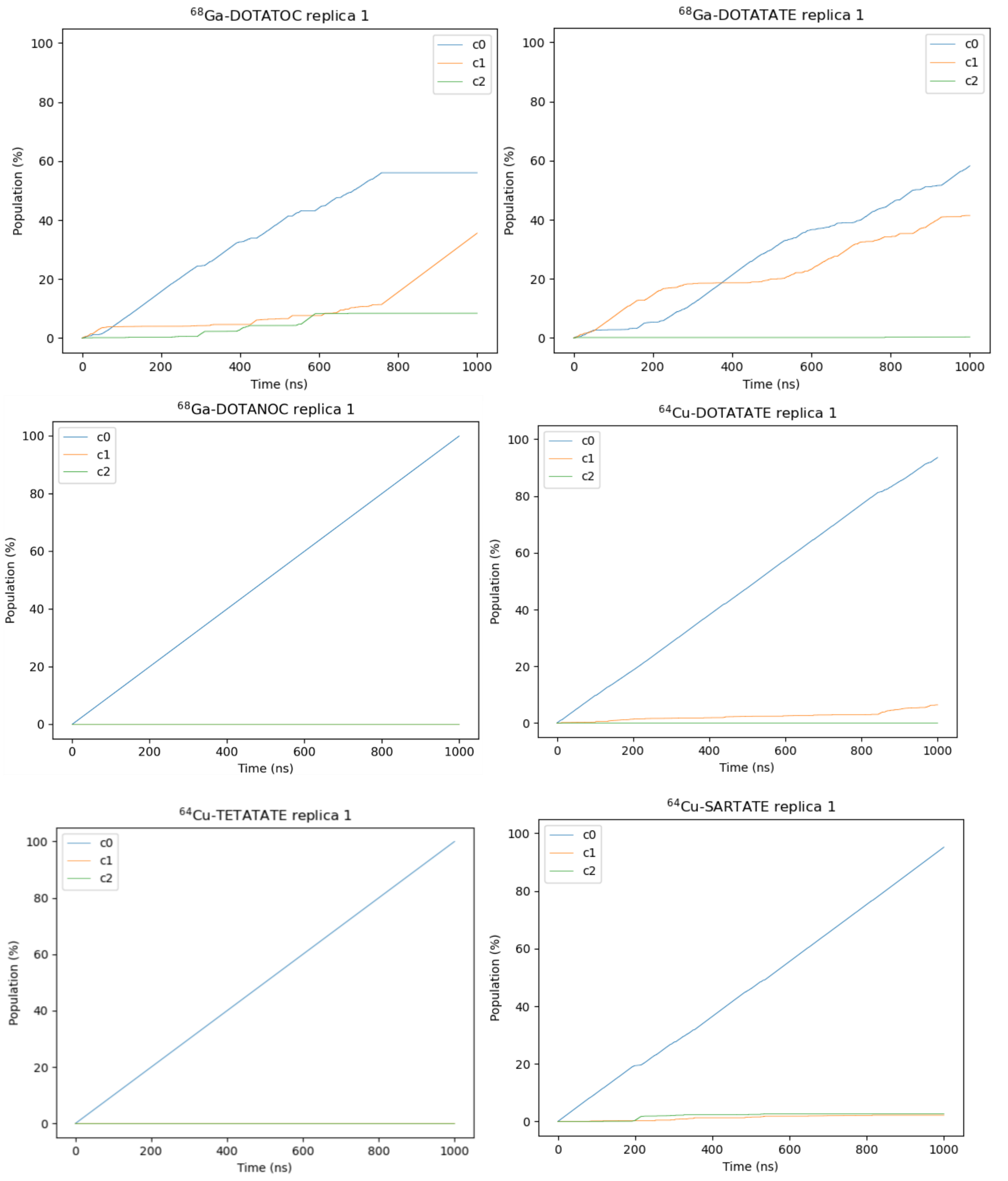

Figure S5. Cluster population over time. We reported the results for one replica of each compound as an exemplification.

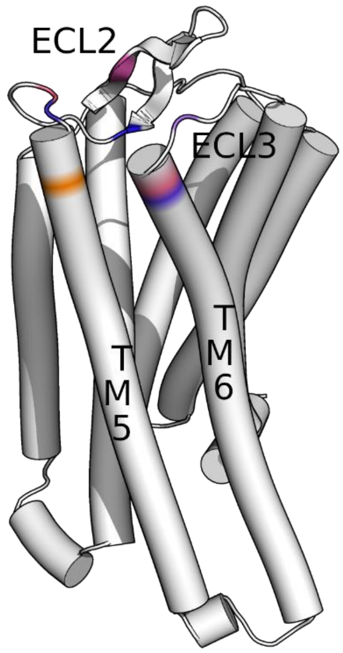

Figure S6. Interaction regions of DOTA. Colors span from blue to yellow, indicating the persistence of interactions (from the lower to the higher). The results of ^{68}Ga-DOTATOC are shown as an exemplification.

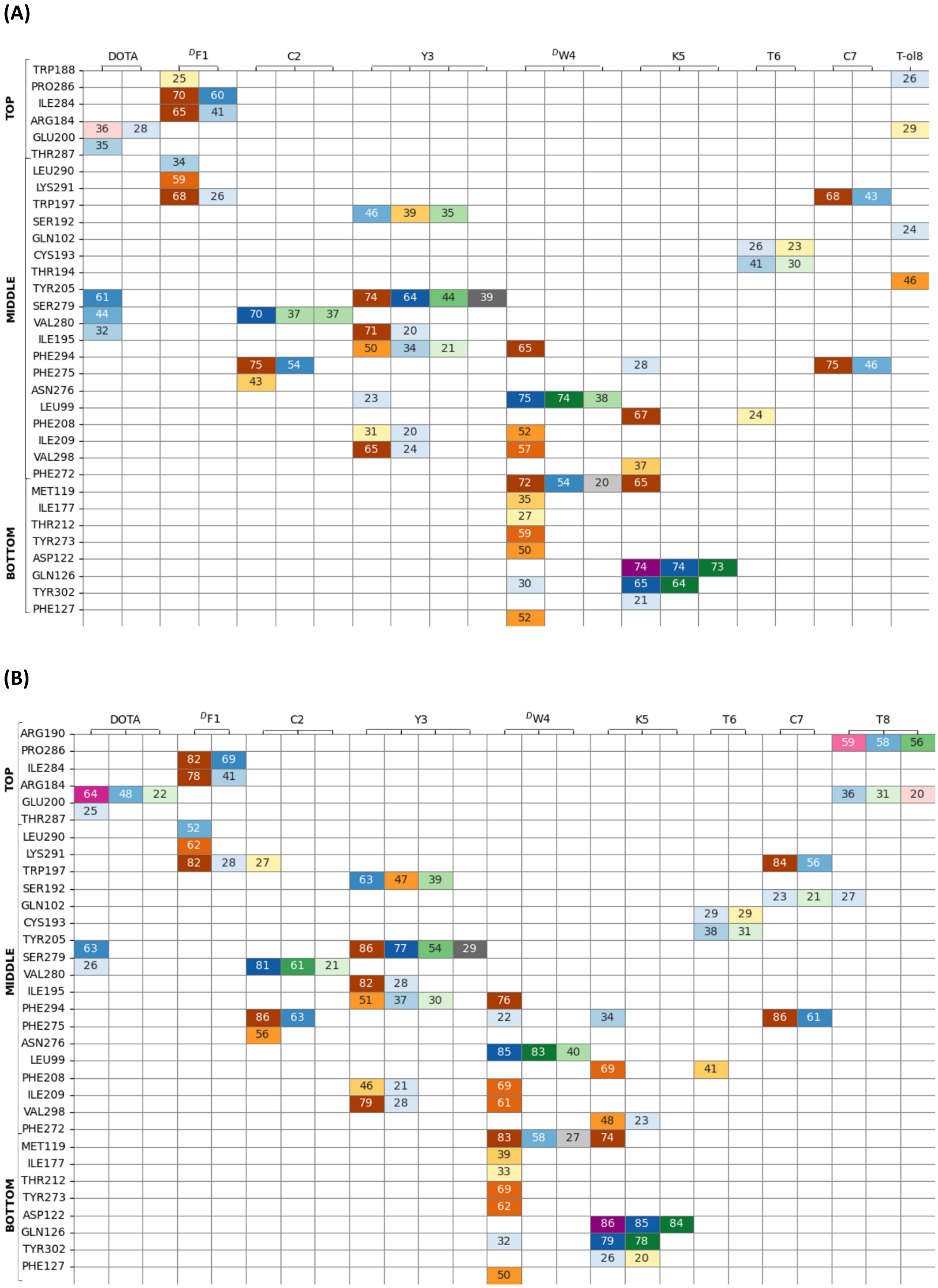
(A)
DOTA
DF1
C2
Y3
DW4
K5
T6
C7
T-ol8
TOP
MIDDLE
BOTTOM
TRP188
PRO286
ILE284
ARG184
GLU200
THR287
LEU290
LYS291
TRP197
SER192
GLN102
CYS193
THR194
TYR205
SER279
VAL280
ILE195
PHE294
PHE275
ASN276
LEU99
PHE208
ILE209
VAL298
PHE272
MET119
ILE177
THR212
TYR273
ASP122
GLN126
TYR302
PHE127
(B)
DOTA
DF1
C2
Y3
DW4
K5
T6
C7
T8
TOP
MIDDLE
BOTTOM
ARG190
PRO286
ILE284
ARG184
GLU200
THR287
LEU290
LYS291
TRP197
SER192
GLN102
CYS193
TYR205
SER279
VAL280
ILE195
PHE294
PHE275
ASN276
LEU99
PHE208
ILE209
VAL298
PHE272
MET119
ILE177
THR212
TYR273
ASP122
GLN126
TYR302
PHE127

(C)

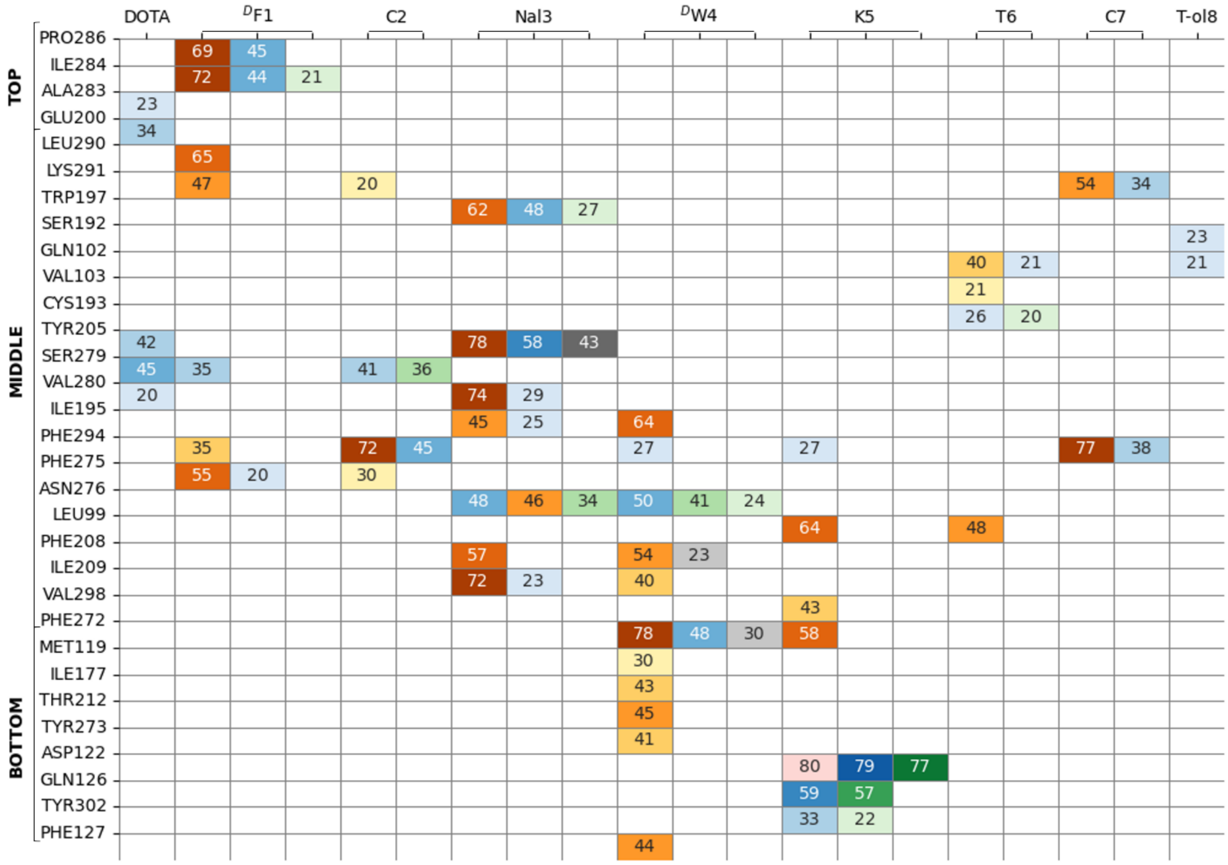

DOTA
DF1
C2
Nal3
DW4
K5
T6
C7
T-ol8
TOP
MIDDLE
BOTTOM
PRO286
ILE284
ALA283
GLU200
LEU290
LYS291
TRP197
SER192
GLN102
VAL103
CYS193
TYR205
SER279
VAL280
ILE195
PHE294
PHE275
ASN276
LEU99
PHE208
ILE209
VAL298
PHE272
MET119
ILE177
THR212
TYR273
ASP122
GLN126
TYR302
PHE127

(D)

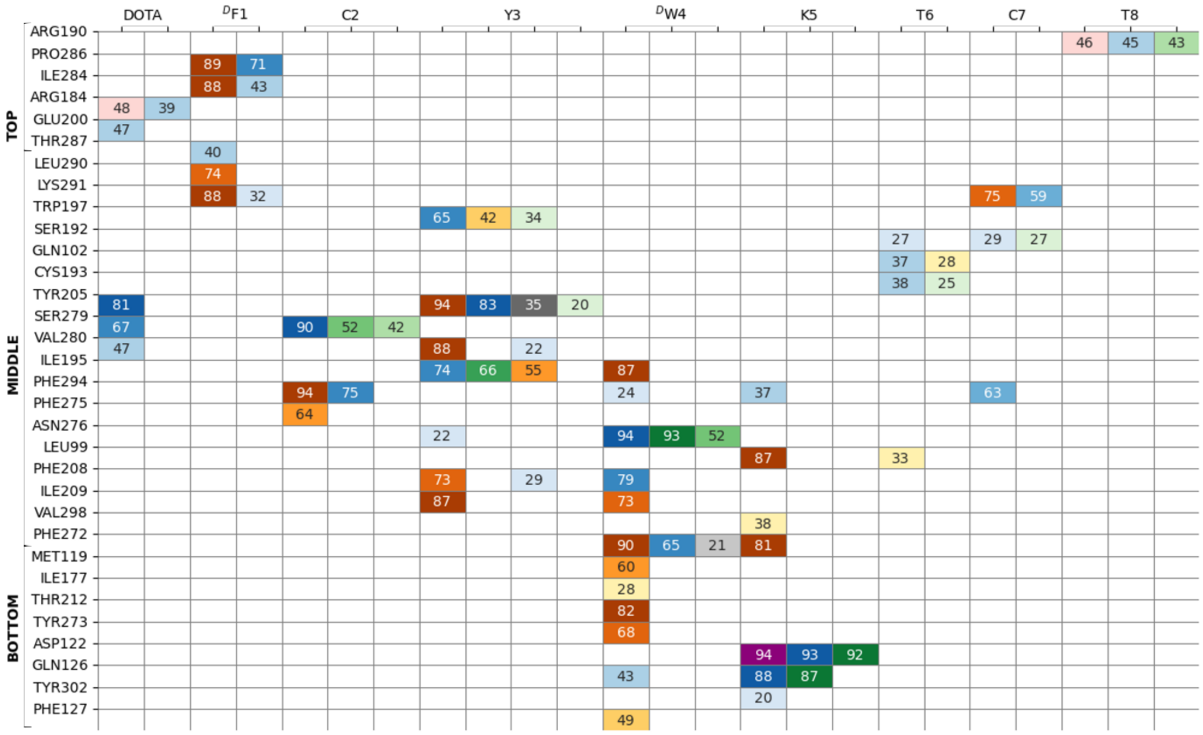

DOTA
DF1
C2
Y3
DW4
K5
T6
C7
T8
TOP
MIDDLE
BOTTOM
ARG190
PRO286
ILE284
ARG184
GLU200
THR287
LEU290
LYS291
TRP197
SER192
GLN102
CYS193
TYR205
SER279
VAL280
ILE195
PHE294
PHE275
ASN276
LEU99
PHE208
ILE209
VAL298
PHE272
MET119
ILE177
THR212
TYR273
ASP122
GLN126
TYR302
PHE127

(E)

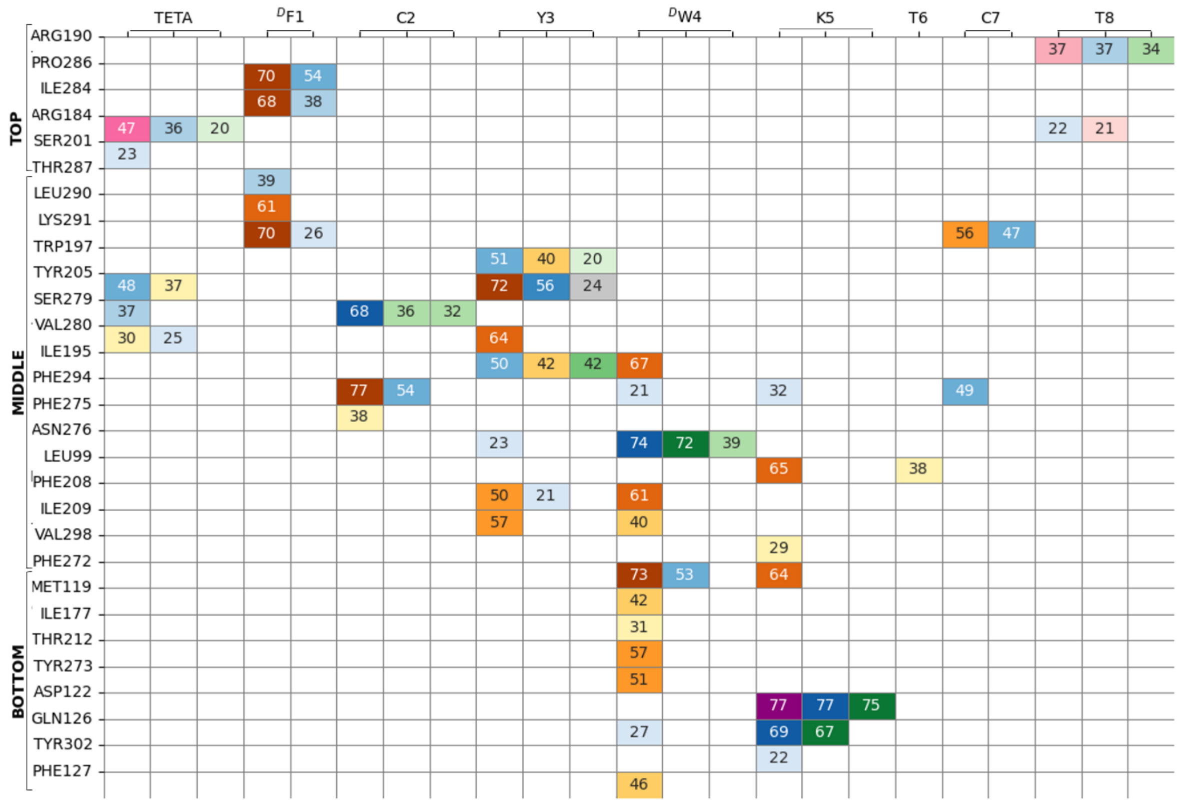

(F)

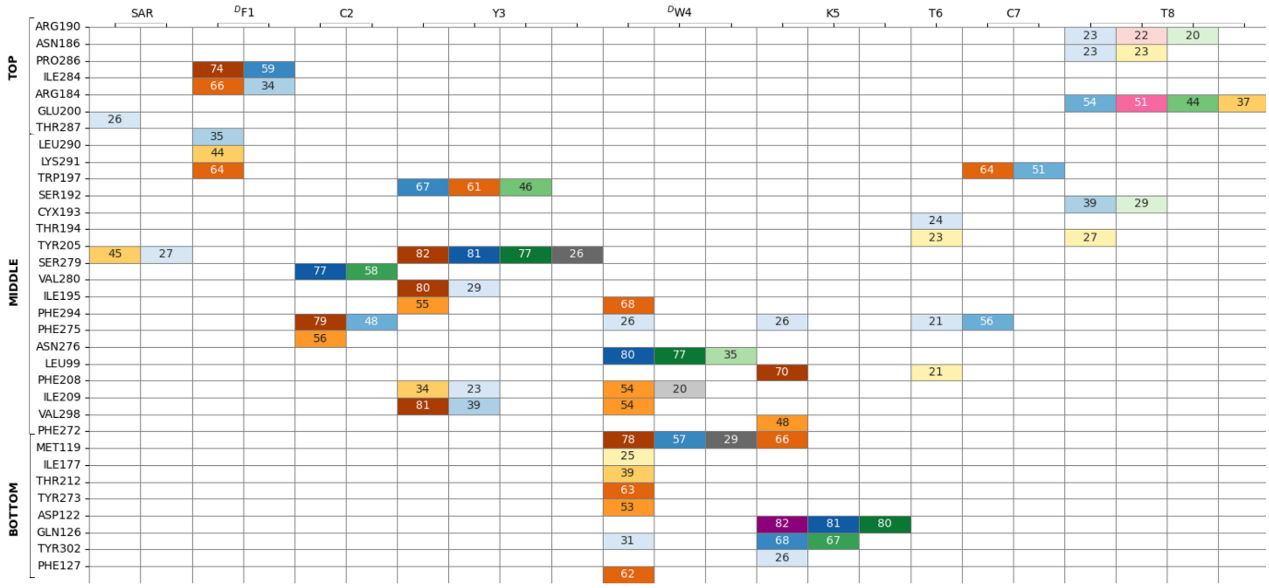

Figure S7. Interaction fingerprint analysis. The values inside the cells report the persistence of the interaction (% of simulation time) between the ligands and SSTR2. **(A)** ^{68}Ga-DOTATOC, **(B)** ^{68}Ga-DOTATATE, **(C)** ^{68}Ga-DOTANOC, **(D)** ^{64}Cu-DOTATATE, **(E)** ^{64}Cu-TETATATE, **(F)** ^{64}Cu-SARTATE. The cells are colored according to the different types of interactions: orange= hydrophobic**,** green= H-bond donor/acceptor, gray= π- π stacking, blue= van der Waals contact, and purple=electrostatic. Only values above the 20% are reported.

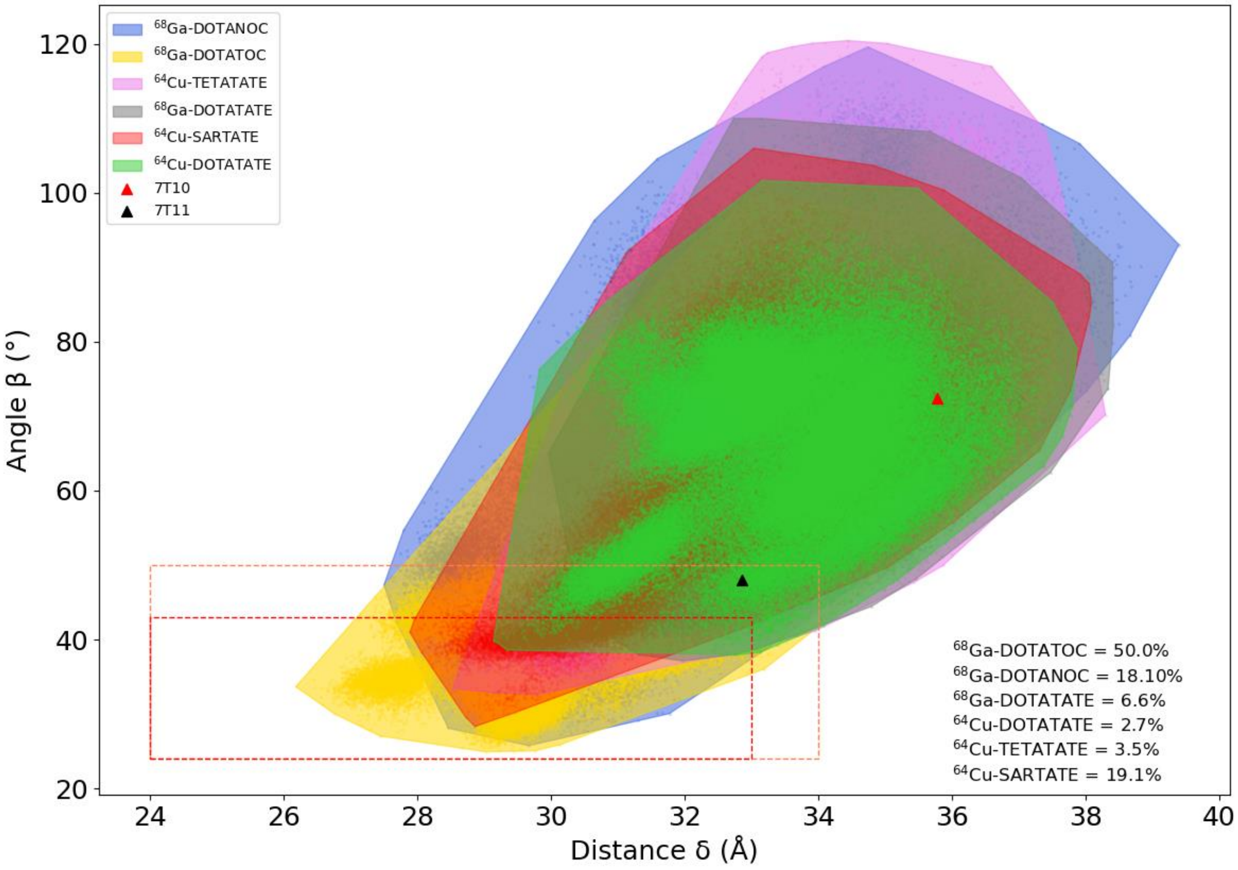

Figure S8. ECL2 opening and closing. Each point of the plot refers to a frame of the MD trajectories. The results of ^{68}Ga-DOTATOC, ^{68}Ga-DOTATATE, ^{68}GA-DOTANOC, ^{64}Cu-DOTATATE, ^{64}Cu-TETATATE, and ^{64}Cu-SARTATE are coloured in yellow, gray, blue, green, magenta, and red, respectively. The red box includes the frames in which the ECL2 is in the closed conformation, the orange box indicates the border between open and closed conformations, according to reference [1]. The areas around dots represent the frame extension. The black triangle represents the starting cryo-EM structure of the receptor (ECL2 in closed conformation, PDB ID 7T11), while the red triangle represents the cryo-EM structure of SSTR2 in complex with somatostatin (ECL2 in open conformation, PDB ID 7T10). Frame percentages in which ECL2 is in the closed conformation are reported at the right bottom of the plot.

Table S2. Comparison of $^{68}Ga^{3+}$/$^{64}Cu^{2+}$ and DOTA/TETA/SAR coordination distances (Å) with respect to experimental data (Exp)[2] and results of different DFT functionals and basis sets. MAE= Mean Absolute Error, PCM= Polarized Continuum Model.

^{68}Ga-DOTA (Connolly surface area: 307 Å^2)

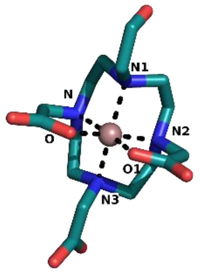

	Exp	B3LYP 6-31G(d,p)	B3LYP 6-31G+(d,p)	B3LYP LANL2DZ
Ga - N	2.1	2.2	2.2	2.3
Ga - N1	2.2	2.1	2.1	2.1
Ga - N2	2.1	2.2	2.2	2.3
Ga - N3	2.2	2.2	2.2	2.2
Ga - O	2.0	1.9	1.9	1.9
Ga - O1	1.9	1.9	1.9	1.9
MAE		0.04	0.05	0.05

^{64}Cu-DOTA (Connolly surface area: 307 Å^2)

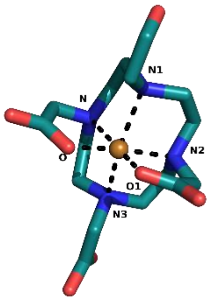

	Exp	B3LYP 6-31G(d,p)	B3LYP 6-31G(d,p) PCM
Cu - N	2.1	2.1	2.1
Cu - N1	2.3	2.5	2.6
Cu - N2	2.1	2.2	2.4
Cu - N3	2.3	2.3	2.4
Cu - O	2.0	2.0	2.0
Cu - O1	2.0	1.9	1.9
MAE		0.05	0.16

^{64}Cu-TETA (Connolly surface area: 320 Å^2)

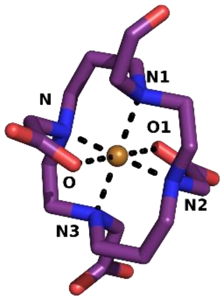

	Exp	B3LYP 6-31G(d,p)	B3LYP 6-31G(d,p) PCM
Cu - N	2.1	2.0	2.0
Cu - N1	2.2	2.3	2.4
Cu - N2	2.1	2.1	2.0
Cu - N3	2.2	2.7	2.6
Cu - O	2.3	2.0	2.0
Cu - O1	2.3	2.0	2.0
MAE		0.21	0.21

^{64}Cu-SAR (Connolly surface area: 381 Å^2)

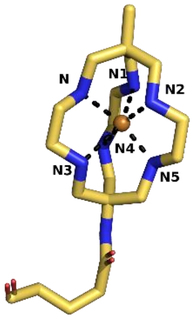

	Exp	B3LYP 6-31G(d,p)	B3LYP 6-31G(d,p) PCM	B3LYP 6-31G+(d,p) PCM
Cu - N	2.3	2.2	2.0	2.0
Cu - N1	2.1	2.0	2.1	2.2
Cu - N2	2.1	2.2	2.4	2.4
Cu - N3	2.1	2.7	2.1	2.1
Cu - N4	2.4	2.1	2.0	2.1
Cu -N5	2.1	2.0	2.4	2.5
MAE		0.20	0.22	0.24

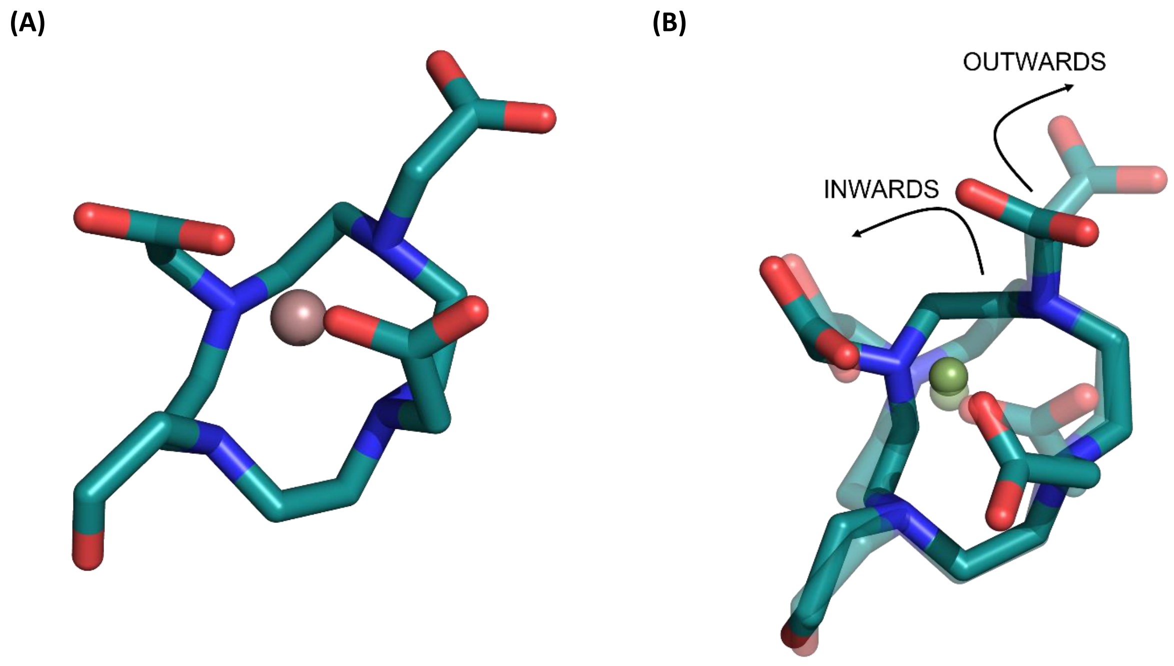

Figure S9. DOTA conformation in (A) ^{68}Ga-DOTATATE and (B) ^{64}Cu-DOTATATE extracted from cluster analysis. All trajectories were concatenated. For ^{68}Ga-DOTA only one geometry has been found, while for ^{64}Cu-DOTA two different geometries have been reported: one with the carboxylic group facing outwards (transparent) and one facing inwards (opaque).

REFERENCES

(1) Gervasoni, S.; Guccione, C.; Fanti, V.; Bosin, A.; Cappellini, G.; Golosio, B.; Ruggerone, P.; Malloci, G. Molecular Simulations of SSTR2 Dynamics and Interaction with Ligands. *Sci. Rep.* **2023**, *13*, 4768. https://doi.org/10.1038/s41598-023-31823-1.

(2) Kubíček, V.; Havlíčková, J.; Kotek, J.; Tircsó, G.; Hermann, P.; Tóth, É.; Lukeš, I. Gallium(III) Complexes of DOTA and DOTA-Monoamide: Kinetic and Thermodynamic Studies. *Inorg. Chem.* **2010**, *49* (23), 10960–10969. https://doi.org/10.1021/ic101378s.
